# Molecular Keys to the *Janthinobacterium* and *Duganella* spp. Interaction with the Plant Pathogen *Fusarium graminearum*

**DOI:** 10.3389/fmicb.2016.01668

**Published:** 2016-10-26

**Authors:** Frederike S. Haack, Anja Poehlein, Cathrin Kröger, Christian A. Voigt, Meike Piepenbring, Helge B. Bode, Rolf Daniel, Wilhelm Schäfer, Wolfgang R. Streit

**Affiliations:** ^1^Department of Microbiology and Biotechnology, Biocenter Klein Flottbek, University of HamburgHamburg, Germany; ^2^Department of Genomic and Applied Microbiology and Goettingen Genomics Laboratory, Institute of Microbiology and Genetics, Georg-August-UniversityGöttingen, Germany; ^3^Department of Molecular Phytopathology, Biocenter Klein Flottbek, University of HamburgHamburg, Germany; ^4^Department of Phytopathology and Biochemistry, Biocenter Klein Flottbek, University of HamburgHamburg, Germany; ^5^Department of Mycology, Goethe University FrankfurtFrankfurt am Main, Germany; ^6^Merck-Stiftungsprofessur für Molekulare Biotechnologie Fachbereich Biowissenschaften and Buchmann Institute for Molecular Life Sciences, Goethe Universität FrankfurtFrankfurt am Main, Germany

**Keywords:** *Janthinobacterium*, *Duganella*, *Fusarium graminearum*, CAI-1, quorum sensing, violacein, bacterium-fungus interaction

## Abstract

*Janthinobacterium* and *Duganella* are well-known for their antifungal effects. Surprisingly, almost nothing is known on molecular aspects involved in the close bacterium-fungus interaction. To better understand this interaction, we established the genomes of 11 *Janthinobacterium* and *Duganella* isolates in combination with phylogenetic and functional analyses of all publicly available genomes. Thereby, we identified a core and pan genome of 1058 and 23,628 genes. All strains encoded secondary metabolite gene clusters and chitinases, both possibly involved in fungal growth suppression. All but one strain carried a single gene cluster involved in the biosynthesis of alpha-hydroxyketone-like autoinducer molecules, designated JAI-1. Genome-wide RNA-seq studies employing the background of two isolates and the corresponding JAI-1 deficient strains identified a set of 45 QS-regulated genes in both isolates. Most regulated genes are characterized by a conserved sequence motif within the promoter region. Among the most strongly regulated genes were secondary metabolite and type VI secretion system gene clusters. Most intriguing, co-incubation studies of *J.* sp. HH102 or its corresponding JAI-1 synthase deletion mutant with the plant pathogen *Fusarium graminearum* provided first evidence of a QS-dependent interaction with this pathogen.

## Introduction

*Janthinobacterium* spp. and bacteria affiliated with the genus *Duganella* are Gram-negative, motile, and aerobic bacteria, which are commonly isolated from soil and aquatic samples. *Janthinobacterium* and *Duganella* spp. are grouped among 11 other genera within the family *Oxalobacteraceae* of the Betaproteobacteria (Lincoln et al., 1999; Baldani et al., 2014). Although most isolates have been obtained from soil or aquatic sites, *Janthinobacterium* can occur in marine habitats in which they can grow to remarkably high titers (Alonso-Sáez et al., 2014). While *Janthinobacterium* as well as *Duganella* appear to be non-pathogenic to humans, animals, and plants, they are well-known for their antifungal effects. For example, *J. livium* suppresses fungal growth on human and amphibian skin or *J. agaricidamnsoum* causes the soft rot disease on the mushroom *Agaricus bisporus* (Becker et al., 2009; Harris et al., 2009; Wiggins et al., 2011; Graupner et al., 2015; Ramsey et al., 2015). The antifungal activities within this *Oxalobacteraceae* family are most likely induced through a regulatory network in response to chitin or degradation products (Cretoiu et al., 2013; Kielak et al., 2013) and the involvement of the secondary metabolite violacein is hypothesized (Brucker et al., 2008; Ramsey et al., 2015).

To date, only few complete or permanent draft genomes of *Janthinobacterium* and *Duganella* are publicly available. In addition, only very few reports have analyzed the mechanisms of cell-cell communication in these genera. We recently reported, that *Janthinobacterium* sp. HH01 (in this study renamed as *Duganella* sp. HH01, Hornung et al., 2013) employs a cell-cell signaling mechanism that was previously only known for *Vibrio* or *Legionella* and is based on the synthesis of α-hydroxyketones. In *V. cholerae* and *L. pneumophila* the corresponding signaling molecule is involved in the regulation of pathogen-host interaction (Tiaden and Hilbi, 2012; Simon et al., 2015) and plays a key role in bacterial competence in *V. cholerae*, which could be linked to the presence of chitin (Lo Scrudato and Blokesch, 2012; Borgeaud et al., 2015). Working on HH01 we have shown that the janthinobacterial autoinducer (JAI-1) is synthesized by the autoinducer synthase JqsA. The *jqsA* gene is encoded within a conserved cluster together with the sensor kinase JqsS and the regulator protein JqsR. We further provided evidence in a previous study that JAI-1 affects the violacein biosynthesis in HH01 (Hornung et al., 2013). In the light of these observations we asked, whether and to which extent *Janthinobacterium* and *Duganella* have established cell-cell communication mechanisms used for intra- and inter-species communication and whether these would be important for their interaction with fungi.

To address these questions we performed comprehensive and combined genome, transcriptome, and mutational analysis of 11 strains. Furthermore, we analyzed the transcriptomes of two *jqsA*-knockout strains using RNA-seq and identified common and highly conserved regulatory quroum sensing (QS) circuits. Additional co-inoculation tests with the plant pathogen *Fusarium graminearum* indicate that the chitin degradation product N-acetyl-D-glucosamine and the QS signal JAI-1 both affect the interaction with the fungus in *Janthinobacterium* but not in *Duganella* isolates.

## Materials and methods

### Bacterial strains and growth

Bacterial strains and plasmids are listed in Table S1. Standard molecular cloning techniques were used (Sambrook and Russell, 2001). Environmental samples were collected from a rainwater-cistern at the Botanical Garden in Klein Flottbek (Hamburg, Germany, HH100–HH107). Samples were enriched on R2A liquid medium (Reasoner and Geldreich, 1985) and purple-pigmented bacteria were repetitively streaked on R2A plates to obtain pure cultures. The same habitat was used to exclude the influence of the isolation source on the impact of the very diverse *Oxalobacteraceae* family on fungi. Strain MP5059B (5059B) was isolated from fruiting bodies of *Ruzenia spermoides*, collected close to the Jakobi Weiher, in the Stadtwald Frankfurt (Germany). Asci were isolated with a sterile needle from perithecia and placed on standard PDA agar (Gams et al., 1998) in an attempt to cultivate the ascomycete. Bacterial strains were grown at 22°C and type strains at 28°C in R2A. Twenty-five μg/ml kanamycin was supplemented when required. 1% (w/v) starch, 4% (w/v) skimmilk or 1% (v/v) tributyrin was added for amylase, protease and lipase activity tests. Cells were grown in M9 without glucose (−G, Elbing and Brent, 2002) and shrimp shell chitin supplementation for 1 week at 22°C to test chitinoclastic activity. Violacein synthesis was measured in TY (Elbing and Brent, 2002). We included genomic data of the strains *J. agaricidamnosum* DSM9628 (HG322949; Lincoln et al., 1999), *J. lividum* strains DSM1522 (De Ley et al., 1978) PAMC 25724 (AHHB00000000; Kim et al., 2012), RIT308 (JFYR00000000; Gan et al., 2014), MTR (JRRH00000000; Valdes et al., 2015), *J*. sp. strains Marseille (CP000269; Audic et al., 2007), RA13 (JQNP01000001; McTaggart et al., 2015), CG3 (APFF00000000; Smith et al., 2013), KBS0711 (LBCO00000000; Shoemaker et al., 2015), Ant5-2 (LNCE00000000; Mojib et al., 2010), *Pseudoduganella violaceinigra* DSM15887 (AUDI00000000; Kämpfer et al., 2012), *D. zoogloeoides* DSM16928 (AX110134; Hiraishi et al., 1997), *D. phyllosphaereae* DSM23865 (Kämpfer et al., 2012), and *D*. sp. HH01 (AMWD00000000; Hornung et al., 2013). Genome information was derived from the Gold data base or IMG (http://www.jgi.doe.gov/): *J*. sp. strains OK676: 60134; 344: 57625, 551a: 57361; NFR18: 57185; B9-8: CP014222; CG23_2: CYSS00000000; and *D*. sp. strains CF402: 61889; OV458: 60275.

### Sequencing, annotation, and bioinformatic tools

For genome sequencing, different methods were used. Strains HH101, HH105, and 5059B were sequenced by a combined approach using the 454 GS-FLX pyrosequencing system (Roche Life Science, Mannheim, Germany) and the Genome Analyzer IIx (Illumina, San Diego, CA, USA), resulting in 2 × 122 bp reads. All other genomes were sequenced on the Genome Analyzer IIx, except for strain HH102 that was sequenced employing the Illumina MiSeq system, resulting in 2 × 301 bp reads. All libraries were prepared according to the protocols of the manufacturers. The Illumina reads were quality filtered; remaining adaptor sequences and the reads with a cutoff phred-33 score of 15 were trimmed using Trimmomatic (Bolger et al., 2014). Illumina reads were assembled using SPAdes genome assembler software (Bankevich et al., 2012) and 454 reads with Newbler 2.8. For the validation of the assembly, QualiMap version 2.1 was used (García-Alcalde et al., 2012). The software tool Prokka (Seemann, 2014) was used for automatic gene prediction and automatic annotation, which was subsequently manually curated by using the IMG-ER system (Markowitz et al., 2014), the Swiss-Prot, TrEMBL, and InterPro databases (Zdobnov and Apweiler, 2001). Genomes were manually analyzed to identify homologs and orthologs of known QS systems, hydrolytic enzymes, secondary metabolites and secretion systems to bypass the bias of automated functional annotations. For Multi locus sequence analysis (MLSA) total protein sequences from the 29 genomes were extracted from the corresponding GenBank files using cds_extractor.pl v0.6 (https://github.com/aleimba/bac-genomics-scripts) and used for downstream analysis with an in-house pipeline at the Goettingen Genomics Laboratory (Poehlein et al., 2015). In detail, proteinortho version 5 (default specification: blast = blastp v2.2.24, *E*-value = 1e-10, alg.-conn. = 0.1, coverage = 0.5, percent_identity = 50, adaptive_similarity = 0.95, inc_pairs = 1, inc_singles = 1, selfblast = 1, unambiguous = 0, Lechner et al., 2011) was used to generate clusters of orthologs groups, inparalogs were removed, MUSCLE (Edgar, 2004) used to align the remaining sequences and poorly aligned positions were automatically filtered from the alignments using Gblocks (Castresana, 2000). A maximum-likelihood tree from 1058 orthologs groups (Table S2) was inferred with 500 bootstraps with RAxML (Stamatakis, 2014). A phylogenetic tree was inferred with neighbor joining and 1000 bootstraps. The script PO_2_MLSA.py is available at github (https://github.com/jvollme). Average nucleotide identity (ANIm) analyses were performed using pyani.py (https://github.com/widdowquinn/pyani). Briefly, nucleotide sequences were extracted from the corresponding GenBank files using seq_format-converter.pl v0.2 (https://github.com/aleimba/bac-genomics-scripts) and subsequently used to run pyani in ANIm mode (uses MUMmer, NUCmer) to align input sequences.

### Mutagenesis of HH102

To obtain HH102Δ*jqsA* (JAB4_14950) PCR products of primers Δ*jqsA*_102_-UP-FP-BamHI, Δ*jqsA*_102_-UP-RP-XbaI, Δ*jqsA*_102_-DS-FP-XbaI and Δ*jqsA*_102_-DS-RP-EcoRI (Table S3) were cloned in the suizide plasmid pNPTS138-R6KT (Lassak et al., 2010). Strain HH102 was transformed by electroporation (Hornung et al., 2013) and single recombinant mutants carrying this construct were selected on R2A. To obtain mutants, heterogenotes were streaked on R2A agar plates supplemented with 10% (w/v) sucrose. Correctness of the deletions was verified with primers oFH106/oFH107 and oFH108/oFH109 (Table S3).

### RNA-Seq analyses—HH01, HH01Δ*jqsA*, HH102, and HH102Δ*jqsA*

Cells were cultivated in 20 ml R2A for 11 h (HH102, HH102Δ*jqsA*) or 13 h (HH01, HH01Δ*jqsA*) and extracted with hot phenol (Aiba et al., 1981). Briefly, cultures were mixed with 25 ml ice-cold killing buffer (20 mM Tris-HCl, pH 7.5, 5 mM MgCl_2_), centrifuged (10 min, 4°C) and shock frozen. Cells were resuspended in 125 μl ice-cold 300 mM sucrose/10 mM sodium acetate (pH 5.2), mixed with 125 μl 2% (w/v) SDS/10 mM sodium acetate (pH 5.2) and incubated (90 s, 65°C). Four hundred microliter hot phenol was added, incubated (3 min, 65°C), frozen and spun down (10 min, RT). Step was repeated. Four hundred microliter phenol:chloroform:isoamylalcohol (50:48:2 v/v) was added twice to the supernatant and spun down. Four hundred microliter chloroform:isoamylalcohol (96:2 v/v) was added and centrifuged (2 min, RT). For RNA precipitation overnight (−20°C) 40 μl 3 M sodium acetate (pH 5.2) and 1 ml 100% (v/v) ethanol were added. RNA was spun down (20 min, 4°C), washed and solved in diethylpyrocarbonate-treated H_2_O. Remaining genomic DNA was removed by digesting with DNAse I (Fermentas, St. Leon-Rot, Germany). The Ribo-Zero magnetic kit (Epicentre Biotechnologies, Madison, WI, USA) was used to reduce the amont of rRNA derived sequences. For sequencing, the strand-specific cDNA libraries were constructed with a NEBNext Ultra directional RNA library preparation kit for Illumina (New England BioLabs, Frankfurt am Main, Germany) and sequenced by using a GAIIx or MiSeq instrument (Illumina Inc., San Diego, CA, USA) in the paired-end mode and running 2 × 75 cycles. Between 28,738,406 and 56,507,330 raw reads were generated for the samples. For quality filtering and removing of remaining adaptor sequences, Trimmomatic (Bolger et al., 2014) and a cutoff phred-33 score of 15 were used. The mapping of the remaining sequences was performed with the Bowtie (version 2) program (Langmead and Salzberg, 2012) using the implemented end-to-end mode, which requires the entire read align from one end to the other. Differential expression analyses were performed with the baySeq program (Mortazavi et al., 2008). Genes with a fold change in expression of ≥2.0, a likelihood value of ≥0.9, and an adjusted *P*-value of ≤ 0.05 (the *P*-value was corrected by the false discovery rate [FDR] on the basis of the Benjamini-Hochberg procedure) were considered differentially expressed. Data set jan4t3 was excluded from calculation. Transcriptomic data were verified with qRT-PCR. Therefore, primer efficiency was tested with a dilution series of genomic DNA using the CFX96 Touch™ Real-Time PCR Detection System (Bio Rad, München, Germany), SYBR Green SuperMix (Quanta Biosciences, Gaithersburg, MD, USA), processed with the Bio-Rad CFX Manager 3.1 software (Bio Rad, München, Germany) and included in the normalized expression (ΔΔCq). Two microgram of DNase treated RNA (RTS DNase™, Mo Bio Laboratories, Inc., Carlsbad, CA, USA) was transcribed in cDNA (SuperScript® VILO cDNA Synthesis Kit and Master Mix, Thermo Scientific, Hampshire, United Kingdom). The genes *rpoD* (HH01, HH102), *dnaG* (HH01), and *dnaB* (HH102) were used as house keeping genes (Table S3). The relative quantity of the three biological samples composed of triplicates are shown in a graph with data relative to control (Figure S1).

### Promoter-fusion studies

Plasmids and primers are listed in Tables S1, S3. Promoter sequence of *vioA* (JAB9_09370, Pvio_107_+JAI-FP-BamHI, and Pvio_107_+JAI-RP-EcoRI) was cloned in pBBR1MCS-2::mCherry (mCherry-FP-EcoRI, mCherry-RP-HindIII with pK18mobII_pKOScvm as template) to obtain pBBR1MCS-2::pvio107+JAI::mCherry. Amplification with Pvio_107_-JAI-FP-XhoI and mCherry-RP-HindIII led to the exclusion of the JAI-1 motif (pBBR1MCS-2::pvio107-JAI::mCherry). Bacterial strains were electroporated as described previously (Hornung et al., 2013).

### Inhibition assay of *Janthinobacterium, Duganella*, and *Fusarium graminearum*

Mycelial plugs of the strain *F. graminearum* 8/1 (Bönnighausen et al., 2015) with a constitutively expressed GFP (Jansen et al., 2005) were taken of a 3-day-old colony grown on complete medium (Leach et al., 1982), placed on R2A plates with 1 × 10^5^ bacterial cells/ml and incubated for 6 days at 22°C. For the liquid assay, 1 × 10^3^ bacterial cells/μl grown in R2A lacking glucose (−G) were mixed with 400 fungal conidia grown on SNA (Nirenberg, 1981) for 1.5–2 weeks, rinsed with ice-cold distilled H_2_O and separated with 100 μm filter. To test the QS effect, 1 × 10^9^ bacterial cells/ml grown in R2A −G supplemented with 0.05% (w/v) G, 10 mM D-glucosamine (DG) or 10 mM N-acetyl-D-gluocosamine (NADG) were filtered, mixed with 400 conidia to a volume of 200 μl in R2A −G and incubated for 72 h at 22°C prior to GFP detection.

### Microscopic analysis of *Janthinobacterium—F. graminearum* interactions

1 × 10^4^ bacterial cells harboring pBBR1MCS-2::P-mCherry were incubated with 400 conidia in R2A –G supplemented with 10 mM NADG for 48 h at 28°C. Subcloning of the promoterless pBBR1MCS-2::mCherry construct using SacI and XhoI endonucleases created the pBBR1MCS-2::P-mCherry plasmid to constitutively express mCherry (Table S1). Micrographs and Z series were captured with the confocal laser-scanning microscope LSM 780 (Zeiss, Germany) by using a Zeiss EC Plan-Neofluar 10 × objective for overview micrographs and a Zeiss C-Aprochromat 63 × water-immersion objective for Z series. First, GFP was excited and detected as described (Ellinger et al., 2013); second, mCherry was excited at 561 nm by using a diode-pumped solid-state (DPSS) laser. Emission filtering was done with a 567–618 nm bandpass filter. Brightfield micrographs were gathered with a transmitted light detector (T-PMT). For image processing the ZEN 2010 (Zeiss) operating software was used.

### Sequence deposition and accession numbers

The whole genome DNA sequences have been deposited at GenBank under the accession numbers LROM00000000 (*D. phyllosphaerae* DSM23865), LRON00000000 (HH101), LRHV00000000 (HH105), *J. lividum* DSM1522 (LRHW00000000), MP5059B (LRHX00000000), HH100 (LRHY00000000), HH102 (LRHZ00000000), HH103 (LRIA00000000), HH104 (LRIB00000000), HH106 (LRIC00000000), and HH107 (LRID00000000). RNA-seq data have been deposited in the SRA archive under the accession number SRP073418 (HH102) and SRP073365 (HH01).

## Results

### *Janthinobacterium* and *Duganella* species suppress growth of the plant pathogen *F. graminearum* in standardized growth assays under laboratory conditions

**Figure 1:**
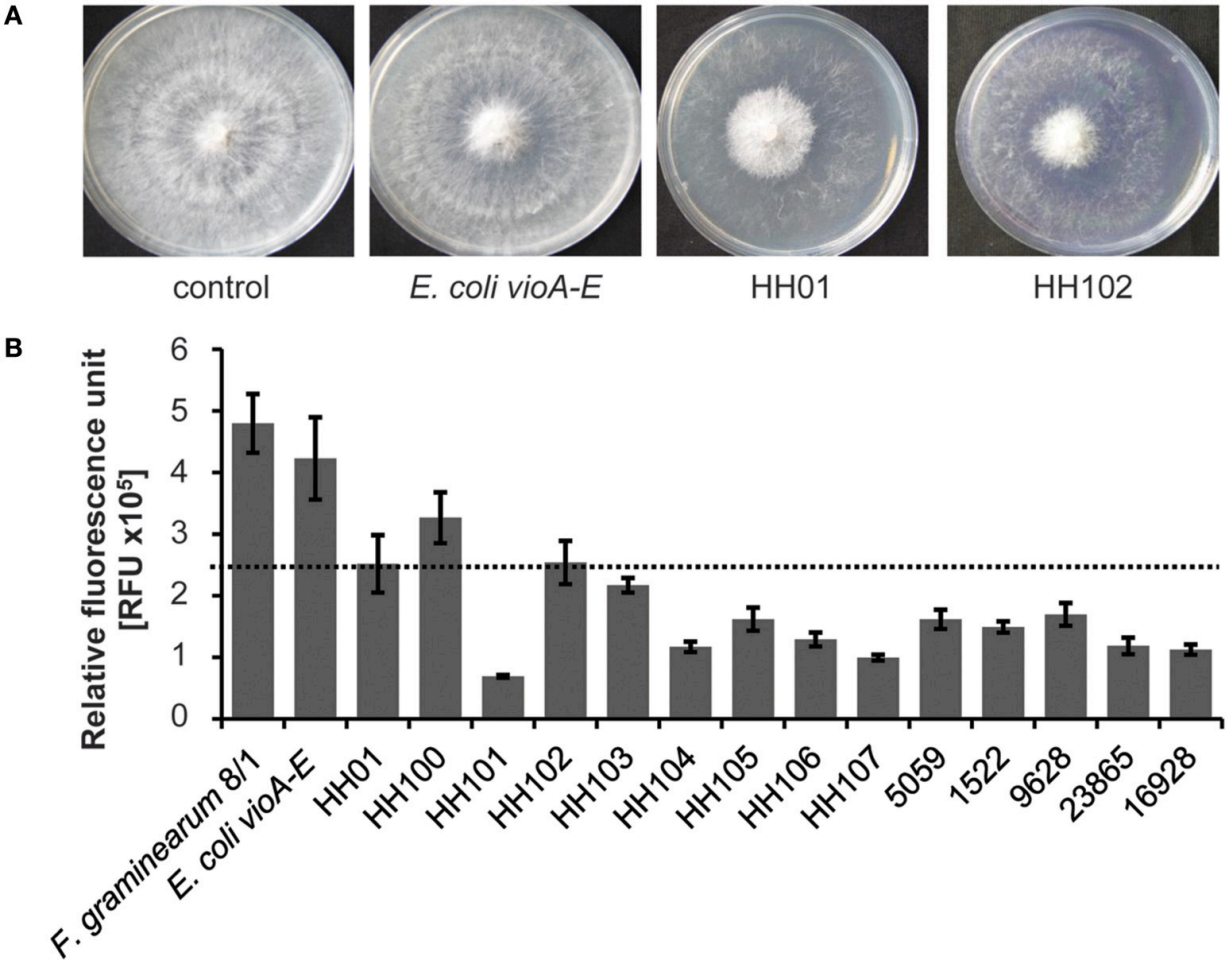
**Inhibition of ***F. graminearum*** 8/1 by isolates and the ***E. coli vioABCDE*** strain**. The *E. coli* strain harbors the violacein *vioABCDE* genes on a pDrive plasmid (Hornung et al., 2013). **(A)** Plate assay to determine the inhibition *of F. graminearum* 8/1 by HH01, HH102, or *E. coli.* Co-incubation studies were performed with 1 × 10^9^ bacterial cells per ml and hyphae of *F. graminearum* 8/1 on solid R2A. The plates were incubated for 6 days at 22°C. Experiments were performed thrice. **(B)** Results of liquid assays to test the *F. graminearum* 8/1 inhibition by the isolates HH100, HH101, HH102, HH103, HH104, HH105, HH106, HH107, and 5059B, the four type strains *J. agaricidamnosum, J. lividum, D. phyllosphaerae* and *D. zoogloeoides*, and *E. coli vioABCDE*. Liquid tests were performed with 180 μl filtered supernatant of 1 × 10^9^ bacterial cells per ml grown in R2A –G and 400 conidia from *F. graminearium* 8/1, solved in 20 μl medium. The assay was incubated for 72 h at 28°C. One out of three independent experiments is shown and each experiment contained four replicates. The error lines indicate standard deviations.

Because of previous reports on antifungal activities of *Janthinobacterium*, we asked the question, whether these inhibitory effects are a common phenomenon of the *Janthinobacterium* and *Duganella* genera within the family *Oxalobacteraceae* and whether this response is linked to QS related processes or the isolation source. To address these questions, we first isolated six *Janthinobacterium* and two *Duganella* species (HH100–HH107) from the same habitat to exclude isolation source-dependent factors and broaden the genetic spectrum of *Oxalobacteraceae*. We included the strains HH01, MP5059B, and the type strains *J. livdium, J. agaricidamnosum, D. phyllosphaerae*, and *D. zoogloeoides* to include diverse environmental habitats, as these strains are derived from aquatic sources, soils, and plant surfaces. The antifungal capacity of all isolates was tested on the well-known plant pathogen *F. graminearum* (Goswami and Kistler, 2004). We established two different assays to assess the antifungal activities. Firstly, we used a growth assay on R2A agar plates and co-incubated fungal and bacterial cells together on these plates (Figure 1A). Secondly, we employed a *gfp*-tagged *F. graminearum* 8/1 variant (Bönnighausen et al., 2015) and monitored its growth based on the fluorescence of the GFP protein in microtiter plates when co-incubated with bacteria (Figure 1B). Since most of the above-named bacterial isolates produce violacein, we also coincubated *F. graminearum* with *E. coli* harboring the violacein synthesis genes *vioABCDE* on a self-replicable plasmid (derived from Hornung et al., 2013, Table S1). This liquid assay allowed a time-dependent monitoring of the fungal response to bacterial supernatants. Both tests implied that all strains affected growth of *F. graminearum* but to different extents (Figures 1A,B). A more than 50% reduction of fungal growth was observed for nearly all isolates (Figure 1B). Further, we observed that the overall fungal growth inhibition was independent of the differing amounts of violacein produced by the different isolates: *D*. *phyllosphaerae* and *D. zoogloeoides* produce no violacein, but display antifungal activities (Table 1, Figures 1A,B). Additionally, we observed that supernatants of *E. coli* clones expressing the *vioABCDE* genes had no major impact on *F. graminearum* growth. These findings indicated that violacein *per-se* was not the primary cause of the fungal growth inhibition. Together with this, we observed that the majority of the strains displayed hydrolytic and chitinoclastic activities, which are possibly involved in fungal defense (Table 1, Schuster et al., 2003; Wagner et al., 2003; Goo et al., 2010).

**Table 1:** Physiological features of the sequenced ***Oxalobacteraceae*** strains and related species^*^.

**Feature**	***Janthinobacterium***									***Duganella***				
	**HH100**	**HH102**	**HH103**	**HH104**	**HH106**	**HH107**	**5059**	**1522**	**9628**	**HH101**	**HH105**	**23865**	**16928**	**HH01**
Violacein synthesis (μg/ml)	1.27 ± 0.12	1.54 ± 0.4	1.19 ± 0.02	1.83 ± 0.08	0.15 ± 0.07	0.93 ± 0.26	0.37 ± 0.02	0.03 ± 0.01	−	6.33 ± 0.15	3.36 ± 0.76	−	−	1.98 ± 0.02
Amylolytic activity	−	−	−	−	−	−	−	−	+	+	+	+	+	+
Lipolytic activity	+	+	+	+	+	+	+	++	+	+++	+++	+++	+	+++
Proteolytic activity	++	++	++	++	++	++	++	−	+	(+)	(+)	−	−	(+)
Chitinoclastic activity	++	++	++	++	++	++	++	++	++	+	+	−	−	+

* *Strains sequenced in this work are indicated by gray shadings. 9628 refers to J. agaricidamnosum, 1522 to J. lividum, 23865 to D. phyllosphaerae and 16928 to D. zoogloeoides. Violacein (μg/ml) was extracted after growth for 24 h in R2A. Amylolytic and proteolytic activities were measured after 72 h of growth on plates and lipolytic activities after 144 h of growth on solid media containing starch, skim milk, or tributyrin. Activities were recorded as diameter of clearing zones around individual colonies. Chitinolytic activity was determined after 1 week of growth in M9 lacking glucose and supplemented with chitin. +, enzymatic was observed; ++, strong activity; +++, very strong activity; (+), weak activity; –, no activity observed, data are mean values of at least three independent biological test; ± indicates the standard deviations*.

### Key traits of the sequenced strains and phylogenetic relationship

**Figure 2:**
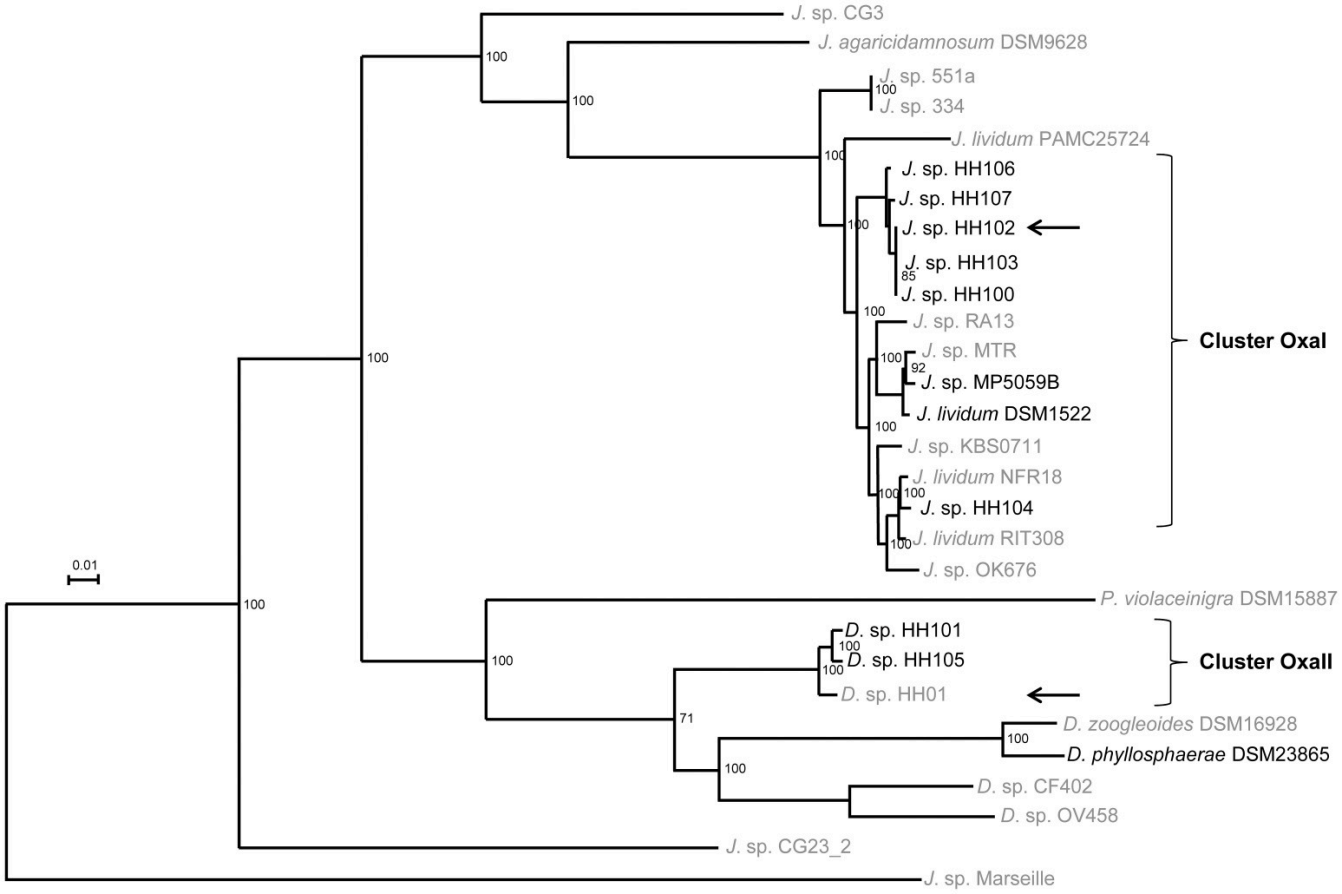
**Multilocus sequence alignment (MLSA) tree**. For Multi locus sequence analysis (MLSA) total protein sequences from the 29 genomes were extracted from the corresponding GenBank files using cds_extractor.pl v0.6 (https://github.com/aleimba/bac-genomics-scripts) and used for downstream analysis with an in-house pipeline at the Goettingen Genomics Laboratory. A maximum-likelihood tree from 1058 orthologs groups (Table S2) was inferred with 500 bootstraps with RAxML (Stamatakis, 2014). A phylogenetic tree was inferred with neighbor joining and 1000 bootstraps. The script PO_2_MLSA.py is available at github (https://github.com/jvollme). Strains sequenced in this study are written in black. The two OxaI (HH102) and OxaII (HH01) representatives are marked with an arrow.

To unravel potential molecular mechanisms involved in bacterium-fungus interaction, we established the genome sequences of the isolated strains and the type strains *J. lividum* and *D. phyllosphaerae* to broaden the genetic spectrum of the *Oxalobacteraceae* family. First, rRNA analysis of the isolates, type strains and *Nitrosomonas europaeae* ATCC25976 (HE862405) classified the strains as phylogenetically associated within the genera *Janthinobacterium* and *Duganella* (data not shown). We included the genomic data of the *J. lividum* strains PAMC 25724 and RIT308 and *J*. sp. strains Marseille, RA13, CG3, OK676, 344, and 551a (Table 2). The genomes ranged from 5.5 to 7.4 Mb and the G+C content from 62.4 to 65.6%. The strains harbored 5467 to 6535 predicted protein-encoding genes, 6 to 32 rRNA genes, and 57 to 82 tRNA genes. The pan- and core-genome of altogether 29 publicly available genome data of *Janthinobacterium, Duganella*, and *Pseudoduganella* genomes comprised of 23,628 and 1058 genes, respectively. Based on the core genome analysis, we constructed a phylogenetic tree using a multilocus sequence analysis (MLSA, Figure 2) as well as the average nucleotide identity (ANIm, Figure S2) to focus on the phylogenetical distribution of the genera *Janthinboacterium* and *Duganella*. Considering these two methods, the isolates HH100, HH102, HH103, HH104, HH106, HH107, and 5059B cluster in proximity to *J. lividum* 1522. Especially HH104, RIT308, and NFR18 are closely related, as well as 5059B, MTR, and *J. lividum* 1522. Based on these findings, the isolates HH100, HH102, HH103, HH104, HH106, HH107, and 5059B are most likely affiliated with the genus *Janthinobacterium.* We designated this cluster as OxaI and HH102 as representative. The strains HH101, HH105, and HH01 cluster within the *Duganella* branch. HH01, HH101, and HH105 are most likely affiliated with the genus *Duganella* and we designated this cluster OxaII. HH01 was selected as representative. Comparative genome analyses indicated that relatively high levels of synteny exist between all analyzed *Janthinobacterium* genomes. Only few regions were identified in the OxaI cluster and *J. lividum* strain that showed relatively high levels of genome divergence (Figure S3A). The analyzed *Duganella* genomes, however, showed higher levels of divergence (Figure S3B). The highest levels of synteny were observed between the *Duganella* strains HH01, HH101, and HH105.

**Table 2:** General genomic features of the strains sequenced in this work and other closely related and sequenced strains^*^.

**Genome traits**	***Janthinobacterium***										
	**HH100**	**HH102**	**HH103**	**HH104**	**HH106**	**HH107**	**5059**	**1522**	**9628**	**Mars**	**25724**
Size (Mbp)	6.7	6.7	6.6	6.4	6.3	5.5	6.4	6.7	5.9	4.1	4.98
G+C content (%)	65.6	62.4	62.5	62.6	62.9	63.0	62.8	62.4	61.0	54.23	60.6
rRNAs	9	8	8	6	8	8	32	11	7	6	21
tRNAs	64	69	73	61	64	66	82	72	73	46	80
Other RNA genes	22	23	22	11	11	14	12	17	0	14	13
Coding genes	5970	5987	5873	5657	5604	5467	5645	5820	5493	3697	4432
With function	4757	4776	4673	4647	4575	4481	4596	4760	4634	2813	3540
%Secondary metabolites	1.04	1.19	1.11	1.39	1.24	1.34	1.41	3.59	8.1	n.d.	n.d.
Scaffolds	150	121	141	65	73	116	100	127	1	1	48
**Genome traits**	***Janthinobacterium***						***Duganella***				
	**RA13**	**CG3**	**OK676**	**344**	**551a**	**RIT308**	**HH101**	**HH105**	**23865**	**16928**	**HH01**
Size (Mbp)	6.4	6.3	6.3	6.4	6.5	6.2	7.4	7.4	6.2	6.3	7.1
G+C content (%)	62.5	65.5	62.8	63.7	63.6	62.8	64.4	64.1	63.9	63.6	64.2
rRNAs	25	15	15	13	9	10	10	24	8	14	20
tRNAs	92	81	65	68	70	83	57	71	58	69	84
Other RNA genes	9	13	14	12	12	−	14	12	14	13	0
Coding genes	5650	5426	5502	5526	5541	5431	6535	6277	5390	5342	5996
With function	4612	4369	4594	4572	4594	4668	5340	5140	4375	4431	4323
%Secondary metabolites	n.d.	n.d.	n.d.	n.d.	n.d.	n.d.	4.21	6.78	1.46	2.33	6.14
Scaffolds	1	7	31	27	28	44	223	89	158	25	2

* *We included genomic data of the strains J. agaricidamnosum (9628), J. lividum strains (1522), PAMC 25724 (25724), RIT308, J. sp. strains Marseille (Mars), RA13, CG3, D. zoogloeoides (16928), D. phyllosphaereae (23865), and HH01. Genome information were derived from the Gold data base or IMG (http://www.jgi.doe.gov/): J. sp. strains OK676, 344, and 551a. HH100–HH107 and MP5059B (5059) refers to the isolates of our lab. Strains sequenced in this work are indicated by gray shadings. “n.d.” refers to not determined and “−“ to not present. Data collection: January 2016*.

### Secondary metabolite encoding potential does not correlate with fungal growth repression

All strains sequenced in this study encoded a variable number of secondary metabolite gene clusters ranging from 1 to 6.8% of the genome (Table 2) with the higher percentages being comparable to well-known secondary metabolite producers like *Streptomyces* or *Myxobacteria* (Bode and Müller, 2005). The identified gene clusters were mostly linked to the biosynthesis of secondary metabolites, including non-ribosomal peptide synthetases (NRPS), and NRPS-polyketide synthases (PKS) hybrid clusters (Medema et al., 2011). Besides NRPS and NRPS/PKS hybrids (HH01, HH101, HH105, *J. agaricidamnosum*, and *J. lividum*), clusters encoding for proteinaceous toxins were observed in all strains. Further, clusters synthesizing terpene class of organic compounds (all strains), a siderophore (*D. phyllosphaerae*), butyrolactone (HH105), hserlactone (*J. lividum*), and a cluster to synthesize the peptides linaridin and thiopeptide-lantipeptides (*J. agaricidamnosum*) were found. Gene clusters coding for compounds linked to the synthesis of pigments (e.g., violacein and aryl-polyenes) were identified. Violacein was synthesized by all strains except for *D. phyllosphaerae* and *D. zoogloeoides* (Table 1), excluding violacein as fungal growth reducing factor. Interestingly, the overall number of genes and ORFs involved in secondary metabolite biosynthesis did not correlate with the observed antifungal effects. However, the presence of proteinaceous toxins and terpenes in all strains indicated a possible involvement in reducing fungal growth.

### *Oxalobacteraceae* harbor the *Vibrio cholerae*- and *Legionella pneumophila*-like QS systems in a conserved cluster as part of the core genome

To better understand, how these bacteria use intra- and inter-species communication to communicate and for the expression and secretion of hydrolytic enzymes and secondary metabolites potentially involved in the effect on *F. graminearum*, the sequenced *Oxalobacteraceae* family affiliated isolates were examined for QS systems present in their genomes. Interestingly, only the *Janthinobacterium* strains 1522, RA13 and CG3 strains harbored *luxI/luxR* homologs genes, potentially involved in the synthesis of N-acyl-homoserine lactones (N-AHL, JALI_51420/JALI_51430, FG13DRAFT_1957/FG13DRAFT_1956, and JANGC3DRAFT_0717/JANGC3DRAFT_0712). Strain 1522 codes for one additional *luxI* homolog (JALI_55550). Several potential *luxR* solos were detected in these strains. It is noteworthy that none of the analyzed strains coded for a *luxS* homolog, necessary for the synthesis of AI-2 (Bassler et al., 1993; Surette et al., 1999). All but one strain addressed in this study code for the JQS system, formerly described in *V. cholerae* and *L. pneumophila* (Tiaden and Hilbi, 2012). The JQS system of HH01 consists of the autoinducer synthase gene *jqsA* (Jab_2c24330), the sensor kinase/phosphatase gene *jqsS* (Jab_2c24340) and the response regulator *jqsR* gene (Jab_2c24350) with the same organization in all strains, but differences in the intergenic region flanking the *jqsA* and *jqsS* genes. This region varied from 42 to 172 bp.

**Figure 3:**
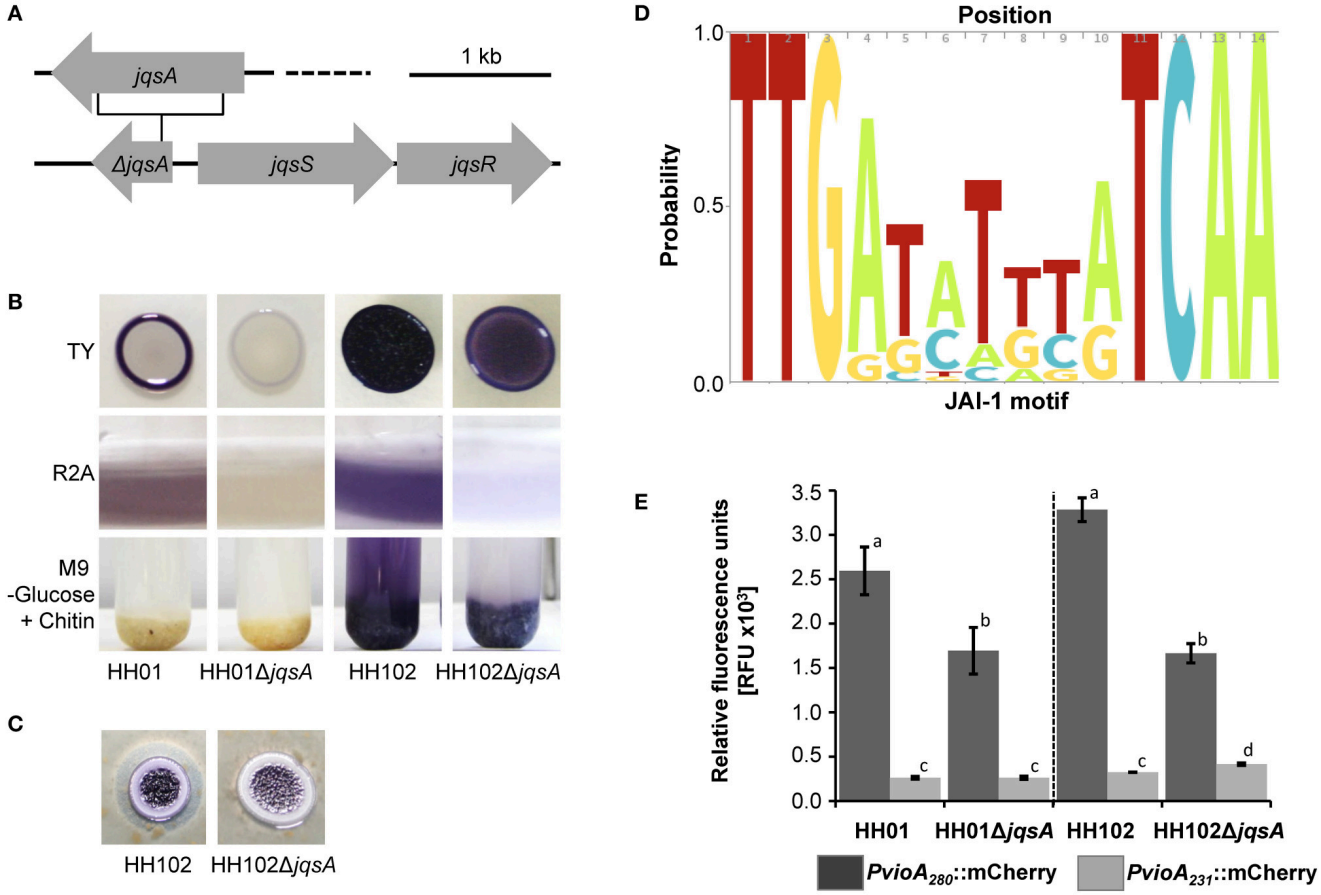
***jqsA* gene deletion of HH01 and HH102, associated phenotypes and influence of the conserved JAI-1 motif**. **(A)** Physical map of the of the JQS cluster encoding the deleted *jqsA* gene and the *jqsS* and *jqsR* genes, including the schematic location of the *jqsA* deletion. **(B)** Growth of HH01, HH01Δ*jqsA*, HH102, and HH102Δ*jqsA* on solid TY media (72 h at 22°C), in liquid R2A media (72 h at 22°C), and in liquid M9 –G, supplemented with chitin (growth for 1 week at 22°C). **(C)** HH102 and HH102Δ*jqsA* on R2A agar plates supplemented with 4% (w/v) skim milk (144 h at 22°C). **(D)** Predicted JAI-1 motif determined by sequence analysis of 28 bp 5′upstream region of the *chiA, jqsA*, and *vioA* genes summarized in Table S5. Sequences extending 14 bp were excluded from analysis. The logo was created using http://skylign.org/ (Wheeler et al., 2014). **(E)** Influence of the JAI-1 motif as part of a 49 bp deletion on the JAI-1 induced mCherry expression. The RFU of the strains HH01, HH102, and their corresponding *jqsA* deletion mutants HH01Δ*jqsA* and HH102Δ*jqsA*, harboring the plasmids pBBR1MCS-2::Pvio_HH107_+JAI::mCherry (dark gray) and pBBR1MCS-2::Pvio_HH107_-JAI::mCherry (bright gray), were determined by measuring the fluorescence at 587/610 nm per OD600 = 1. In pBBR1MCS-2::Pvio_HH107_-JAI::mCherry a 49 bp region was deleted containing the complete JAI-1 motif. The cells were grown for 24 h at 22°C in R2A media and RFUs measured. Error bars indicate the standard deviations. Data are values of a minimum of three tests. Bars carrying the same letters of HH01/HH01Δ*jqsA* or HH102/HH102Δ*jqsA* are statistically not significantly different using *P*-values of < 0.05 (students *t*-test).

To further examine the importance of the *jqsA* gene for QS-dependent regulation in the family *Oxalobacteraceae*, we constructed a deletion mutant of the *jqsA* gene of strain HH102 (HH102Δ*jqsA*) as OxaI representative and used the strain HH01Δ*jqsA* as OxaII representative (Hornung et al., 2013). Mutants were verified by DNA sequencing and complemented with functional AI synthases (Figure S4), whereby HH01Δ*jqsA* had previously been complemented (Hornung et al., 2013). A deletion of the *jqsA* gene resulted in HH102 in a strong reduction of the violacein production. This was most pronounced when grown in liquid R2A or on solid TY (Figure 3B). A further phenotype associated with the *jqsA* mutation included an altered violacein production in response to chitin embedment for strain HH102 (Figure 3B). Thereby, planctonic cells and chitin-attached cells of the wild type HH102 produced violacein. Interestingly, HH102Δ*jqsA* cells synthesized violacein only when attached to chitin, indicating a possible regulatory role of chitin. This effect was not observed for HH01 or its *jqsA* deletion mutant. Additionally, in HH102Δ*jqsA* the proteolytic activity was attenuated compared to the parent strain (Figure 3C).

### RNA-Seq identifies few QS-regulated genes in HH01 and HH102 and a conserved JAI-1 motif in the promoter region of QS regulated genes

Based on the results, we analyzed the global gene expression patterns of the strains HH01 and HH102 and their corresponding JAI-1 synthase mutants at the transition from exponential to stationary growth phase. We chose the early time point, because at the onset of the stationary growth phase many of QS-dependent processes are turned on. For each sample a minimum of 19.6 million cDNA reads could be uniquely mapped to the reference genomes. In the comparative analysis of RNA-seq data we considered genes with a log fold-change of ≥ 2.0, a likelihood value of ≥ 0.9, and a FDR-value of ≤ 0.05 as statistically significant. Expression analysis by qRT-PCR technology was used to confirm part of the RNA-seq data (Table 3). In HH01 a total set of 31 genes was differentially expressed and JAI-1 affects the expression of 14 in HH102 (Table 3). The HH01 QS-regulated genes were mainly organized in gene clusters that could be linked to four functional classes: secondary metabolite biosynthesis, lipopolysaccharide biosynthesis, a short chain dehydrogenase cluster and a cluster coding for a novel transport system. In HH102 two gene clusters and five single genes were differentially regulated. This included one out of two encoded type VI secretion system (T6SS) clusters and one cluster necessary to assemble a Flp pilus. With the exception of the secondary metabolite violacein no shared homologs genes of HH01 and HH102 were QS regulated, despite the core genome affiliation (Table 3).

**Table 3:** QS-dependent genes identified in HH01 and HH102 using RNA-seq and qRT-PCR^*^.

**Locus tag**	**Possible function**	**Log-fold change**	**Phylogenetic classification**	**JAI-1 motif**	**qRT-PCR (fold change)**
**HH01**					
**Transport System**					
Jab_2c10580^*^	Outer membrane protein, *oprM*	−1.71	I + II + J	y	
Jab_2c10590	HlyD family secretion protein	−2.28	I + II + J		−1.77
Jab_2c10600^*^	ATPase	−1.09	I + II + J		
Jab_2c10610	Inner membrane transport permease, *yhhJ*	−2.37	I + II + J		
**Lipopolysaccharide Synthesis**					
Jab_2c07990	Lipopolysaccharide biosynthesis protein	−2.14	II + J	y	−3.92
Jab_2c08000	Putative tyrosine-protein kinase	−2	II + J		
Jab_2c08010^*^	Hypothetical protein	−1.97	II + J		
Jab_2c08020^*^	UDP-N-acetylglucosamine 2-epimerase, *wecB*	−1.95			
Jab_2c08030	Polysaccharide deacetylase	−2.21	II + J		
Jab_2c08040	Hypothetical protein	−2.09	II + J		
Jab_2c08050^*^	Glycosyl transferase	−1.97	II + J		
Jab_2c08060^*^	Eight transmembrane protein, *epsH*	−1.94	II + J		
Jab_2c08070	Possible glycosyl transferase	−2.42	II + J		
Jab_2c08080^*^	Possible asparagine synthetase	−1.9	II		
Jab_2c08090^*^	Possible glycosyltransferase	−1.69	II + J		
Jab_2c08100^*^	Putative capsular polysaccharide biosynthesis protein	−1.94	II + J		
**Secondary Metabolites**					
Jab_2c08810	L-tryptophan oxidase, *vioA*	−2.61	I + II + J	y	−5.97
Jab_2c08820	Violacein biosynthesis protein, *vioB*	−2.41	I + II + J		
Jab_2c08830	Monooxygenase, *vioC*	−2.32	I + II + J		
Jab_2c08840	Tryptophan hydroxylase, *vioD*	−2.34	I + II + J		
Jab_2c08850	Violacein biosynthesis protein, *vioE*	−2.45	I + II + J		
Jab_2c08860	Predicted arabinose efflux permease	−2.54	I + II + J		
Jab_2c16610	Kynureninase, *kynU*	+2.67	I + II − HH105	y	+5.4
Jab_2c16620	Tryptophan 2,3-dioxygenase, *kynA*	+2.4	I + II − HH101		
Jab_2c35330^*^	Hypothetical protein	−1.18	U	n.a.	
Jab_2c35360	Possible polyketide synthase	−2.39	U		
Jab_2c35370	Possible homoserine O-succinyltransferase, *metA*	−3.68	U		
Jab_2c35380	Non-ribosomal peptide synthetase/amino acid adenylation protein	−3.34	U		
Jab_2c35390	Predicted glycine/serine hydroxymethyltransferase, *glyA*	−3.04	U		
Jab_2c35400	Non-ribosomal peptide synthetase	−3.49	U		−7.76
Jab_2c35410	Non-ribosomal peptide synthetase	−3.68	U		
**Sdr Cluster**					
Jab_2c26560	Predicted aldo/keto reductase	−7.77	U	y	
Jab_2c26580	Predicted short-chain dehydrogenase/reductase	−8.77	II		−353.35
Jab_2c26590	Predicted transcriptional regulator AraC family	−3.96	II		
Jab_2c26600^*^	Hypothetical protein	−1.14	II		
**Other**					
Jab_1c10070	Putative serine/threonine protein kinase	−2.48	II	n	
Jab_1c13730	Predicted porin	−2.24	I + II − HH104	n	
Jab_1c18190	Putative polysaccharide deacetylase	+2.3	II	y	
Jab_1c09990	Tetracycline resistance protein	−3.18	I + II − HH104	y	
Jab_2c08460	Predicted histidine kinase	−2.17	I + II + D +J	y	
Jab_2c08470	Predicted diguanylat cyclase	−2.21	I + II + D +J		
Jab_2c19720	TIR domain containing protein	−2.29	C − Marseille	y	
**HH102**					
**Type VI Secretion System**					
Jab4_02740^*^	Type VI secretion system protein, *impL*	−3.91	I, II, D, J-1522	y	
Jab4_02750^*^	Type VI secretion system protein, *impM*	−3.29	C −M-Pd		
Jab4_02760^*^	OmpA-OmpF porin, OOP family	−4.22	C −M-Pd		
Jab4_02770^*^	Type VI secretion system protein, *impK*	−2.53	I, II, D-16928, J	y	
Jab4_02780^*^	Type VI secretion system protein, *impJ*	−2.09	C −M		
Jab4_02790^*^	Type VI secretion system protein, *vasD*	−2.12	I, 01, J-9628		
Jab4_02800^*^	Hypothetical protein	−3.73	C −M	y	
Jab4_02810^*^	Type VI secretion system protein, *impB*	−3.4	C −M,-9628		
Jab4_02820	Type VI secretion system protein, *impC*	−3.64	C −M		
Jab4_02830^*^	Type VI secretion system secreted protein, *hcp*	−3.99	C −M		
Jab4_02840	Type VI secretion system protein, *impF*	−3.44	C −M		
Jab4_02850	Type VI secretion system protein, *vasG*	−3.44	C −M, J-9628		−13.86
Jab4_02860^*^	Type VI secretion system protein, *impA*	−3.35	C −M		
**Flp Pilus Assembly**					
Jab4_35690	Flp pilus assembly protein, *flp/pilA*	−2.58	I, J	y	−5.07
Jab4_35700^*^	Prepilin peptidase, *cpaA*	−2.5	C −M		
Jab4_35710^*^	Hypothetical protein	−3.07	C −M		
Jab4_35720	Flp pilus assembly protein, *cpaB*	−3.21	C −M		
Jab4_35730^*^	Flp pilus assembly protein, *cpaC*	−3.01	C −M. −CG23		
Jab4_35740^*^	Hypothetical protein	−3.13	I, J		
Jab4_35750^*^	Tight adherence protein, *tadG*	−3.02	C −M		
Jab4_35760^*^	Tight adherence protein, *tadE*-like	−3.58	I, J		
Jab4_35770	Tight adherence protein, *tadE*-like	−3.56	C −M		
Jab4_35780^*^	Flp pilus assembly protein, *cpaE*	−3.35	I −5059, II, D, J-1522		
Jab4_35790	Flp pilus assembly protein, *cpaF*	−3.6	C		
Jab4_35800^*^	Tight adherence protein, *tadB*	−3.49	C −M		−10.53
Jab4_35810^*^	Tight adherence protein, *tadC*	−3.37	C −M		
Jab4_35820^*^	TPR repeat-containing protein	−3.22	I, J		
**Secondary Metabolites**					
Jab4_20480^*^	Inner membrane transport protein YdhP	−1.76	I + II + J	y	
Jab4_20490^*^	Hypothetical protein	−1.7	I + II + J		
Jab4_20500^*^	Tryptophan hydroxylase, *vioD*	−1.79	I + II + J		
Jab4_20510^*^	Monooxygenase, *vioC*	−1.84	I + II + J		
Jab4_20520	Violacein biosynthesis protein, *vioB*	−2.09	I + II + J		
Jab4_20530	L-tryptophan oxidase, *vioA*	−2.22	I + II + J		−6.48
**Others**					
JAB4_03960^*^	Predicted phospholipase	−2.9	I—5059	n	
JAB4_13490	Hypothetical protein	−2.6	I, J	y	
JAB4_13500^*^	Hypothetical protein	−3.22	I, J		
JAB4_16300	PRC-barrel domain protein	−2.67	I, II, J, D	y	−6.25
JAB4_23200	Hemerythrin-like metal-binding domain protein	−2.58	I, II, J-9628	y	
JAB4_30510	Response regulator receiver domain-containing protein	−2.52	I, J-9628	y	
Jab4_42080	Predicted aminoglycoside 3-N-acetyltransferase	−2.89	I, J-9628	y	−3.57
Jab4_42090^*^	Hypothetical protein	−3.21	I, J-9628		
Jab4_42320	Hypothetical protein	−2.81	I, J	n	
Jab4_42330^*^	Hypothetical protein	−2.83	I, J		
JAB4_54620^*^	Predicted soluble aldose sugar dehydrogenase	−3.04	I, J-9628	y	

* We considered genes with a fold-change of ≥ 2.0, a likelihood value ≥ 0.9 and a FDR-value of ≤ 0.05 as statistically significant. Genes that did not match these criteria but were part of an operon and confirmed by qRT-PCR are indicated with^*^. y refers to the presence of the JAI-1 motif, n to no JAI-1 motif identified; n.a., not available as the promoter lies within a contig border. For phylogenetic classification, isolates are classified according to the OxaI (I) or OxaII (II) affiliation and type strains were abbreviated with J (Janthinobacterium) and D (Duganella). 9628 refers to J. agaricidamnosum, 1522 to J. lividum, 23865 to D. phyllosphaerae, 16928 to D. zoogloeoides, 5059 to J. sp. MP5059B, M to J. sp. Marseille and PD to P. violaceinigra. − indicates the lack of this gene within the genome of the following strain, C refers to the core genome and U to the unique presence within this strain. Genes/ORFs being part of the same gene cluster or operon as indicated by consecutive locus tags are either light or dark gray shaded.

Intrigued by these observations, we asked, whether a common regulatory motif would be located upstream of all the QS regulated genes. Comparing the 5′ region of the differentially expressed genes in HH01 and HH102, we identified a conserved palindromic sequence in the promoter region of almost all regulated genes and operons (Table 3, Table S4). This conserved sequence consisted of 14/15 nucleotides with the sequence TTGA_N6/7_TCAA (Figure 3D) and was located 243 bp upstream of the presumed *vioA* translational start site. This motif was also observed upstream of all *vioA, jqsA*, and *chiA* genes of the strains sequenced and analyzed in this work (Table S5). Analyzing the motif position of the JAI-1 regulated genes (Table S4), the position ranged from 80 to 339 bp upstream of the translational start sites. To further verify the importance of this motif we constructed a promoter fusion carrying the *vioA* promoter within a 280 bp fragment fused to a mCherry reporter gene on a self-replicable plasmid (Table S1). This fusion was mobilized into HH01 and HH102 and the corresponding *jqsA* mutants. As expected, it was active in the parent strains and two-fold less active in the *jqsA* mutants. Deletion of the TTGA_*N*6/7_TCAA motif as part of a 49 bp deletion resulted in a 75–90% reduction of promoter activity compared to the strains carrying the complete *vioA* promoter (Figure 3E). These observations indicate a regulatory role of this conserved sequence with respect to the JAI-1-dependent gene expression. Thus, we designated this TTGA_N6/7_TCAA sequence JAI-1 motif. However, next to JAI-1 the chitin derivate DG and NADG affect the *vioA* promoter controlled mCherry expression in dependency on the JAI-1 motif as well (data not shown). This suggests a complex regulatory circuit between QS and chitin metabolism and might explain why a discrepancy between the wild type strains harboring the construct lacking the JAI-1 motif and the corresponding gene deletion mutants with the JAI-1 motif was observed.

### HH102Δ*jqsA* is attenuated in its virulence against the plant pathogen *F. graminearum*

**Figure 4:**
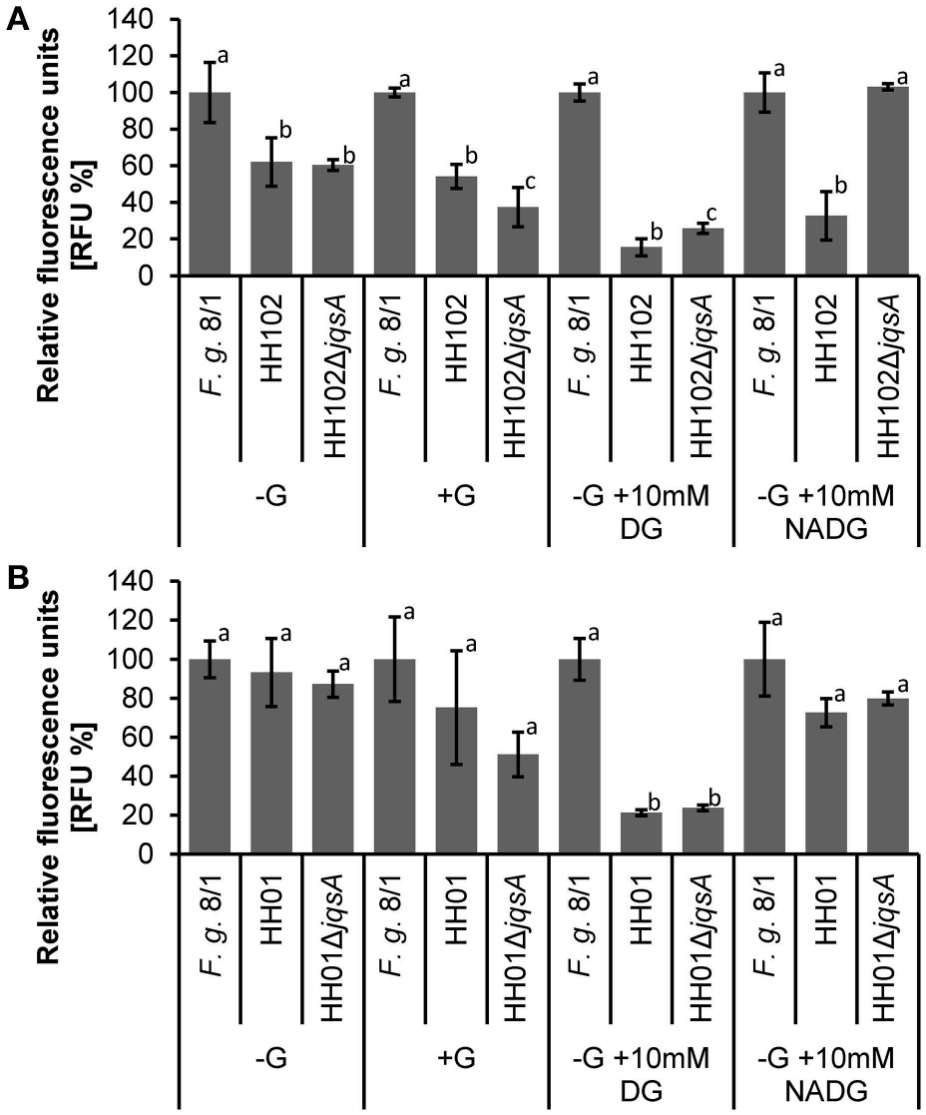
**Bacterial inhibition of ***F. graminearum*** growth in a microtiter plate assay**. The HH102, HH01, HH102Δ*jqsA*, and HH01Δ*jqsA* cells were grown in R2A −G. Media were supplemented with 0.05% (w/v) glucose (G), 10 mM D-glucosamine (DG), or 10 mM N-acetyl-D-glucosamine (NADG) and cells were incubated at 22°C for 24 h. One-hundred and eighty microliter of filtered supernatant (1 × 10^9^ cells per ml) and 400 fungal conidia were incubated in a volume of 200 μl for 72 h at 28°C, shaking. Expression of chromosomal integrated GFP in *F. graminerarum* 8/1 was detected at 485/20; 528/20 nm. GFP expression of *F. graminearum* 8/1 in different media was based on *F. graminearum* 8/1 grown in R2A −G. This is set as 100%. Herein, fold changes are calculated setting each *F. graminearum* media control as 100% and calculating the relative fluorescence of co-incubated *F. graminearum* with bacteria for the respective medium. Experiments were performed at least four-times with four replicates. Bars carrying the same letters within each condition are statistically not significantly different using *P*-values of < 0.05 (students *t*-test). One individual experiment of **(A)** HH102/HH102Δ*jqsA* and **(B)** HH01/HH01Δ*jqsA* is shown.

**Figure 5:**
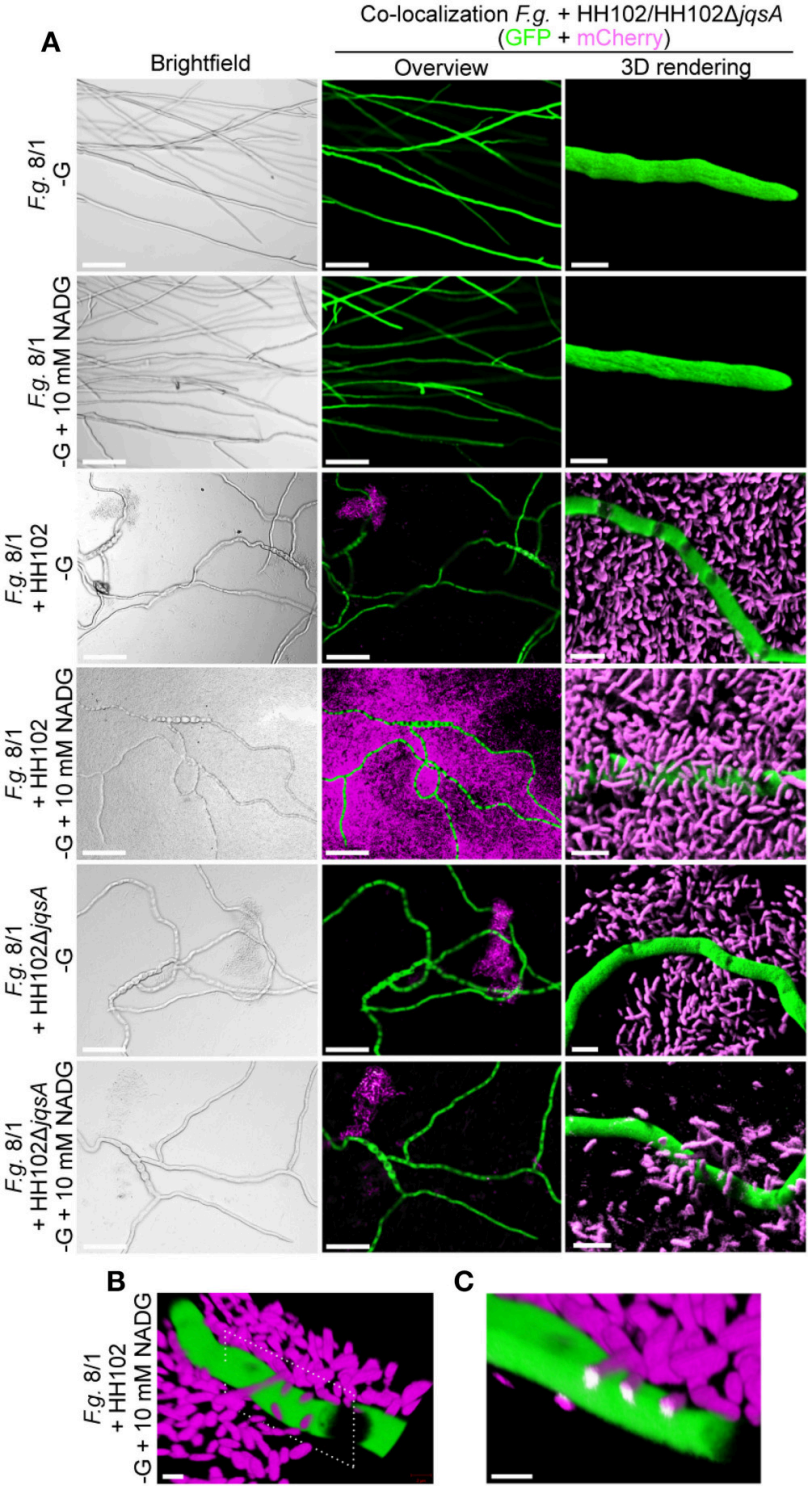
**Microscopic images reflecting the interaction between HH102 or HH102Δ***jqsA*** and ***F. graminearum***. (A)** Co-localization of mCherry-tagged HH102 and HH102Δ*jqsA* cells and GFP-tagged *F. graminearum* in co-cultures. Left panels, bright field overview images; middle panels, same section image as on left side panel but using fluorescence detection for GFP and mCherry; and right panels, 3D rendering of selected regions. Scale bars: overview (left and middle panels) micrographs = 100 μm; 3D-rendered micrographs = 5 μm (right panels). No mCherry-based fluorescence was visible in control cultures without bacteria. **(B)** 3D-projection of HH102 cells attached to hyphae of *F. graminearum* in cultures supplemented with NADG. Dotted frame indicates plane of *in silico* cross section in **(C)**. Scale bar = 2 μm. **(C)** *In silico* cross section at sites of bacterial attachment on *F. graminearum* hyphae. Bacterial interaction and penetration into fungal hypha is indicated by white color. Scale bar = 2 μm. The HH102 and HH102Δ*jqsA* cells constitutively expressing mCherry were co-incubated with *F. graminearum* expressing GFP in 200 μl R2A −G supplemented with or without 10 mM N-acetyl-D-glucosamine (NADG) at 28°C for 72 h. Micrographs were taken by confocal laser-scanning microcopy. Green color shows GFP-, magenta color shows mCherry-emitted fluorescence.

**Figure 6:**
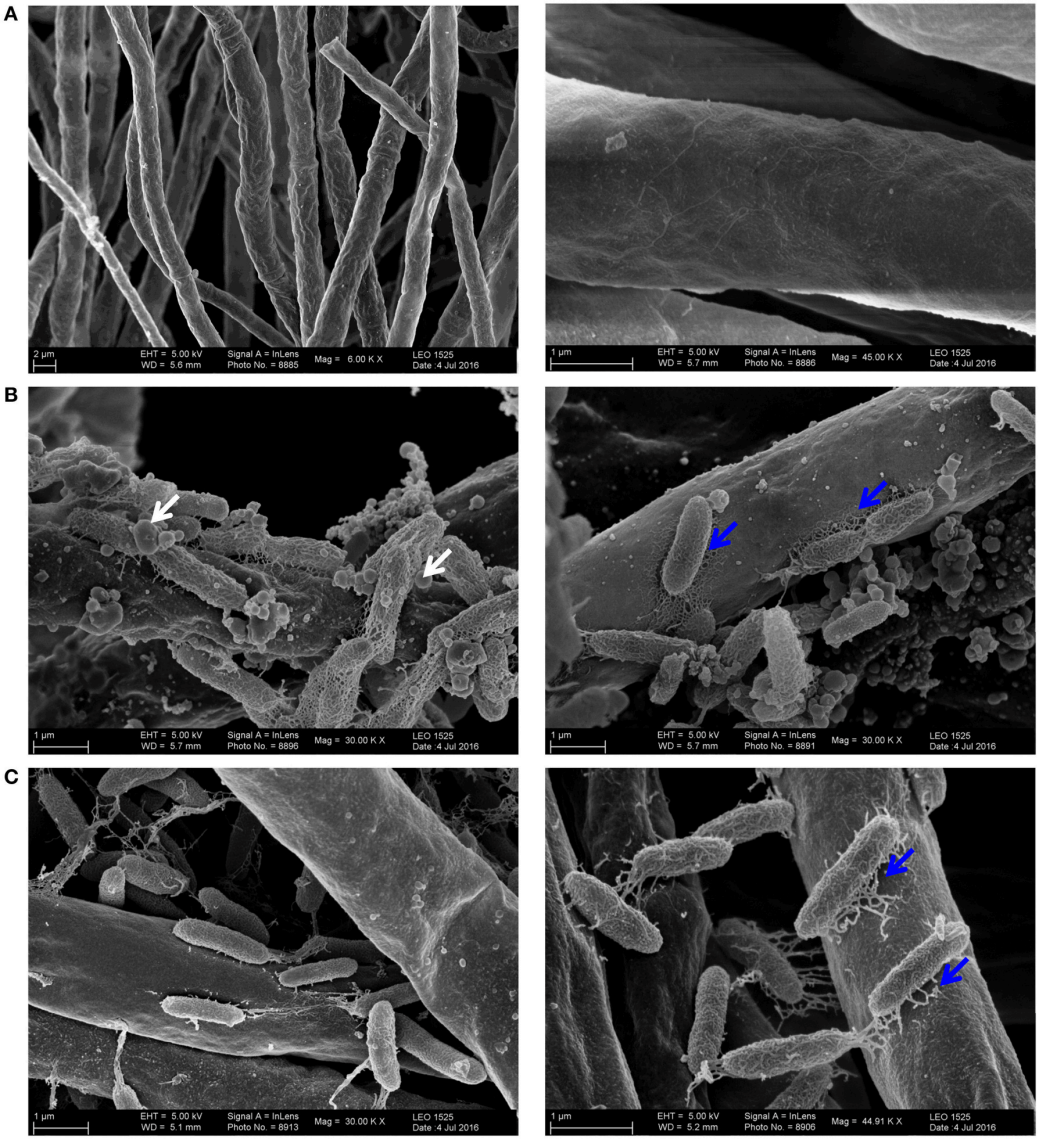
**Scanning electron microscopic images reflecting the interaction between HH102 or HH102Δ***jqsA*** and ***F. graminearum*****. **(A)** Uninfected hyphae growing linear; **(B)** HH102 colonizing fungal hypae OMVs are indicated with white arrows in the left hand panel and netlike structures attaching the cells to the fungal hyphae are indicated in the right hand panel with blue arrows; **(C)** HH102Δ*jqsA* colonizing fungal hypae with less dense and differentially structured netlike structures and lacking OMVs. Maginfications used are indicated in the individual images. The HH102 and HH102Δ*jqsA* cells were pretreated for 24 h at 22°C with 10 mM N-acetyl-D-glucosamine (NADG) and co-incubated with *F. graminearum* expressing GFP in 200 μl R2A −G supplemented with 10 mM NADG at 28°C for 72 h.

In the light of these observations we asked, whether the JAI-1 dependent gene expression and signaling circuit would affect the interaction of HH01 or HH102 with *F. graminearum*. To address this question we assayed the inhibition of fungal growth caused through culture supernatants of both bacterial strains and their corresponding *jqsA* mutants (Figures 4A,B). We chose culture supernatants, because we hypothesized that the bacteria would release growth inhibitory compounds into the surrounding medium in a QS-dependent manner. Therefore, filtered bacterial supernatants were co-incubated with *F. graminearum* for 72 h in microtiter plates as described in “Material and Methods” and fungal growth was recorded as relative fluorescence (RFU). Further, we preincubated the bacterial cells with either glucose (G), D-glucosamine (DG) or N-acetyl-D-glucosamine (NADG) to test whether these chitin degradation products have impact on the overall bacterial response. As expected culture supernatants obtained from the parent strains of HH102 and HH01 inhibited fungal growth (Figures 4A,B). Interestingly, HH102 and HH01 strongly inhibited fungal growth when supernatants of HH102 or HH01 were employed that were derived from cultures grown in the presence of DG. The lack of the *jqsA* gene, however, did not significantly affect growth inhibition in the mutants compared to the parent strains, when supernatants of cells were employed that had been grown in the presence of DG. When HH102 or HH102Δ*jqsA* were grown in the presence of 10 mM NADG, the growth inhibition of the fungus differed strongly between wild type and the corresponding mutant. Under these conditions HH102Δ*jqsA* was 2.5 ± 0.3-fold less active against *F. graminearum* (Figure 4A). Interestingly, this difference in fungal growth inhibition was only observed in the presence of culture supernatants obtained from cells that had been grown in the presence of NADG but not DG or G (Figure 4A). These inhibition data correlated well with microscopic analyses of the interaction between *F. graminearum* and the bacterial strains HH102 or HH102Δ*jqsA* (Figure 5). In general, the presence of both bacterial strains caused a reduced germination rate of the fungal conidia and twisted growth of hyphae with irregular lateral branches (Figure 5A, four lower panels and Figure 6A). In contrast and in the absence of bacteria, the hyphae grew straight and uni-directional (Figure 5A, two upper panels). In these tests HH102 formed dense biofilms or cell assemblages around hyphae of *F. graminearum* (Figure 5A, mid section panels). The hyphae were often completely covered and enclosed by the bacteria. Interestingly, the presence of NADG appeared to promote a direct attachment of HH102 cells on hyphae of *F. graminearum* (Figures 5B,C). Furthermore, the HH102Δ*jqsA* strain formed less dense and looser biofilms around the hyphae (Figure 5A, four lower panels). This observation was supported by images obtained using scanning electron microcopy. Thereby, several intriguing observations could be made. First, wild type strain HH102 secreted outer membrane vesicles (OMVs, Figure 6B, white arrows) and second, HH102 attached tightly to fungal hyphae by forming netlike structures around the bacterial cells. Interestingly, OMVs were less frequently observed in mutant cells. Further, the netlike structures attaching mutant cells to hyphae surfaces appeared different and less dense (Figure 6C). Altogether these data imply that the JAI-1-dependent signaling is involved in the interaction with the fungus and that it attenuates the growth suppression in the presence of NADG. Our data further suggest that DG and NADG interfere with this rather complex regulatory circuit.

## Discussion

Today our knowledge on bacterium-fungus interactions is sparse and only now the first reports are appearing that give insight into this important form of microbe-microbe interaction (Kobayashi and Crouch, 2009; Frey-Klett et al., 2011; Lackner et al., 2011; Stopnisek et al., 2016). Within these settings, we have established the genomes of 11 bacteria affiliated with the genera of *Janthinobacterium* and *Duganella* to get insight into the bacterial mechanism employed for cell-cell communication and the interaction with the fungus *F. graminearum*. We produced, by far, the largest and most comprehensive dataset for this bacterial group. Using these data we constructed a detailed MLSA tree (Figure 2). This MLS analysis indicated that *Janthinobacterium* and *Duganella* strains form a polyphyletic group within the familiy *Oxalobacteraceae*. The majority of the analyzed genomes cluster into two distinct phylogenetic groups, which we designated OxaI and OxaII. The core genome of the 29 *Oxalobacteraceae* strains consisted of 1058 genes and the pan genome of 23,628 genes, indicating that there is a high degree of variation within this family. This reflects the ability of the species to adapt to a wide range of environmental niches. Even though complete and draft genomes were used for core/pan genome analysis, the use of draft genomes had no effect on the size of the core and pan genome. All draft genomes contained contigs larger than 0.5 kb and gaps in the draft genomes represent repetitive regions such as rRNA clusters, transposases, or phage regions which are mainly covered by small contigs (0.5–5 kb).

Within this study, we provide evidence that all tested isolates code for antifungal properties, regardless of the phylogenetic affiliation to the genus *Janthinobacterium* or *Duganella*. These are most likely secondary metabolites, bacterial hydrolases and other “factors” secreted to attack the fungus (Tables 1, 2) and as suggested for the *Oxalobacteraceae* genus *Collimonas* (Song et al., 2015). The occurrence of secondary metabolite biosynthesis gene clusters appears to be a common trait of *Janthinobacterium* and *Duganella* species (Table 2). However, the overall number of genes coding for secondary metabolites is highly variable within both genera and ranges from 1 to 8% of all predicted protein-encoding genes (Table 2). It is noteworthy that almost all strains with the exception of the two *Duganella* type strains code for the biosynthesis of violacein. However, we further provide strong evidence that this antifungal effect is not primarily linked to the synthesis of violacein *per-se*, but is also affiliated to other not yet identified proteinaceous factors (Figures 1A,B, Table 1), as the fungal growth reducing effect is evened out by heating bacterial supernatants (data not shown). Interestingly, while the violacein biosynthesis genes are organized in the same order and in a conserved operon as reported for *Chromobacterium violaceum*, the expression regulation of the *vioABCDE* genes appears to be different. Instead of a N-AHL-dependent regulatory circuit of *C. violaceum* the violacein biosynthesis in *Janthinobacterium* and *Duganella* is partly regulated through a CAI-1/LAI-1-like molecule (Morohoshi et al., 2010; Stauff and Bassler, 2011; Hornung et al., 2013). This signaling pathway has been identified in *V. cholera* and uses a (*S*)-3-hydroxytridecan-4-one autoinducer molecule (CAI-1) for communication. In *V. cholera*, CAI-1 is involved in the repression of virulence and plays an important role in biofilm dissolution (Zhu and Mekalanos, 2003; Higgins et al., 2007). It has also been shown that CAI-1 plays a major role in natural competence (Lo Scrudato and Blokesch, 2013), as well as the structurally similar *L. pneumophila* LAI-1 molecule (3-hydroxypentadecan-4-one). LAI-1 promotes pathogen-host cell interactions, the production of virulence factors and controls the change of the *L. pneumophila* life cycle from the replicative to the transmissive growth phase. Interestingly, LAI-1 is directly involved in inter-kingdom signaling, as a lack of LAI-1 modulates migration of eukaryotic cells (Spirig et al., 2008; Tiaden et al., 2010; Simon et al., 2015; Schell et al., 2016). Moreover, *V. harveyi* uses amongst others (Z)-3-aminoundec-2-en-4-one as CAI-1 molecule to regulate luminescence, for example (Henke and Bassler, 2004). Most remarkably, the genes linked to the synthesis of α-hydroxyketone-like AI molecules were identified in all analyzed *Janthinobacterium* and *Duganella* isolates, but *J*. sp. Marseille. The JQS system consists of the designated AI synthase *jqsA*, adjacent to the cognate sensor kinase *jqsS* and the regulator *jqsR* (Figure 3A).

In our study, we provided experimental evidence that JAI-1-like signaling affects the violacein biosynthesis in HH01 and HH102 and influences protease secretion in HH102 (Figure 3C). Furthermore, RNA-seq analyses implied that 31 genes are controlled through JAI-1-dependent signaling in HH01 and 14 in HH102 (Table 3). Most of the JAI-1-dependent genes were organized in clusters or putative operons. Interestingly, the majority of the QS-regulated genes carried a highly conserved motif in the 5′ direction of the transcriptional start site (Figure 3D). The frequent occurrence of this motif suggests a common regulatory principle for all these genes. We named this TTGA_N6/7_TCAA sequence JAI-1 motif. Analyzing the RNA-seq data, the regulation of genes for the biosynthesis of secondary metabolites, T6SS, and Flp-Pilus assembly are of special interest. Within this framework the biosynthesis of secondary metabolites of *Collimonas* was suggested to affect fungal growth (Song et al., 2015), the T6SS of *V. cholerae* acts together with chitin and CAI-1 as competence pheromone to function as predatory killing device (Borgeaud et al., 2015) and the T4P of *V. vulnificus* was implied to be necessary for the attachment to chitin (Williams et al., 2015). Testing the wild type strain HH102 and the *jqsA* gene deletion mutant HH102Δ*jqsA* in a BFI with *F. graminearum*, one of the most intriguing observations in this study is that the lack of the *jqsA* gene resulted in a decreased fungal growth inhibition (Figure 4A). The observation is remarkable, because this α-hydroxyketone-synthesizing QS system has previously not been reported to be involved in bacterial-fungal interactions. Further, the observation that the JAI-1 dependent fungal growth inhibition also depends on the presence of the chitin degradation product NADG, implies a role of chitin in the *Janthinobacterium*-specific QS regulatory circuit. NADG is a chitin degradation product mostly occurring in aquatic microorganisms (Hillman et al., 1989; Gooday, 1990). As the expression of chitinases is induced by chitin degradation products (Beier and Bertilsson, 2011), we suggest that the bacteria mostly attached to fungal hyphae express and secrete chitinases in response to NADG. Therefore, we conclude that JQS and NADG are involved in reducing fungal growth. This includes the JAI-1-regulated T6SS and T4P expression, both interfering with the chitin metabolism and fungal growth suppression (Borgeaud et al., 2015; Williams et al., 2015) and the QS- and NADG-induced exopolysaccharide expression. Further contributing factors might be the QS-independent expressed secondary metabolites. Altogether, our observation is in line with work reported for *V. cholerae*, which induces natural competence in the presence of chitin and CAI-1 and with work describing an enhanced chitin degrading activity in a pathogen suppressive environment (Lo Scrudato and Blokesch, 2013; Dalia et al., 2014). Thus, the observation that the lack of the JAI-1 signaling molecule affects fungal growth inhibition implies an involvement of the janthinobacterial AI in the interaction with eucaryotic organisms. Future work has to unravel this complex regulatory circuit.

## Author contributions

Conceived and designed the experiments: FH, CK, and WRS. Performed the experiments: FH, CK, AP, and CV. Analyzed the data: FH, AP, and WRS. Contributed reagents/materials/analysis tools: RD, WS, WRS, CV, and MP. Wrote the paper: FH and WRS. Edited the paper: FH, WRS, CV, RD, WS, HB, and MP.

## Funding

This work was supported in part by the BMBF grants 0315586F and 0315587A to WRS.

### Conflict of interest statement

The authors declare that the research was conducted in the absence of any commercial or financial relationships that could be construed as a potential conflict of interest.

## References

[B1] Aiba H. Adhya S. de Crombrugghe B. (1981). Evidence for two functional gal promoters in intact *Escherichia coli* cells. J. Biol. Chem. 256, 11905–11910. 6271763

[B2] Alikhan N. F. Petty N. K. Ben Zakour N. L. Beatson S. A. (2011). BLAST ring image generator (BRIG): simple prokaryote genome comparisons. BMC Genomics 12:402. 10.1186/1471-2164-12-402 21824423 PMC3163573

[B3] Alonso-Sáez L. Zeder M. Harding T. Pernthaler J. Lovejoy C. Bertilsson S.. (2014). Winter bloom of a rare betaproteobacterium in the Arctic Ocean. Front. Microbiol. 5:425. 10.3389/fmicb.2014.00425 25191307 PMC4138443

[B4] Audic S. Robert C. Campagna B. Parinello H. Claverie J. M. Raoult D.. (2007). Genome analysis of *Minibacterium massiliensis* highlights the convergent evolution of water-living bacteria. PLoS Genet. 3:e138 10.1371/journal.pgen.0030138 17722982 PMC1950954

[B5] Baldani J. I. Rouws L. Cruz L. M. Olivares F. L. Schmid M. Hartmann A. (2014). The family *Oxalobacteraceae*, in The Prokaryotes - Alphaproteobacteria and Betaproteobacteria, eds Rosenverg E. DeLong E. F. Lory S. Stackebrandt E. Thompson F. (Berlin; Heidelberg: Springer-Verlag), 919–974. 10.1007/978-3-642-30197-1_291

[B6] Bankevich A. Nurk S. Antipov D. Gurevich A. A. Dvorkin M. Kulikov A. S.. (2012). SPAdes: a new genome assembly algorithm and its applications to single-cell sequencing. J. Comput. Biol. 19, 455–477. 10.1089/cmb.2012.0021 22506599 PMC3342519

[B7] Bassler B. L. Wright M. Showalter R. E. Silverman M. R. (1993). Intercellular signalling in *Vibrio harveyi*: sequence and function of genes regulating expression of luminescence. Mol. Microbiol. 9, 773–786. 10.1111/j.1365-2958.1993.tb01737.x 8231809

[B8] Becker M. H. Brucker R. M. Schwantes C. R. Harris R. N. Minbiole K. P. (2009). The bacterially produced metabolite violacein is associated with survival of amphibians infected with a lethal fungus. Appl. Environ. Microbiol. 75, 6635–6638. 10.1128/AEM.01294-09 19717627 PMC2772424

[B9] Beier S. Bertilsson S. (2011). Uncoupling of chitinase activity and uptake of hydrolysis products in freshwater bacterioplankton. Limnol. Oceanogr. 56, 1179–1188. 10.4319/lo.2011.56.4.1179

[B10] Bode H. B. Müller R. (2005). The impact of bacterial genomics on natural product research. Angew. Chem. Int. Ed. Engl. 44, 6828–6846. 10.1002/anie.200501080 16249991

[B11] Bolger A. M. Lohse M. Usadel B. (2014). Trimmomatic: a flexible trimmer for Illumina sequence data. Bioinformatics 30, 2114–2120. 10.1093/bioinformatics/btu170 24695404 PMC4103590

[B12] Bönnighausen J. Gebhard D. Kröger C. Hadeler B. Tumforde T. Lieberei R.. (2015). Disruption of the GABA shunt affects mitochondrial respiration and virulence in the cereal pathogen *Fusarium graminearum*. Mol. Microbiol. 98, 1115–1132. 10.1111/mmi.13203 26305050

[B13] Borgeaud S. Metzger L. C. Scrignari T. Blokesch M. (2015). The type VI secretion system of *Vibrio cholerae* fosters horizontal gene transfer. Science 347, 63–67. 10.1126/science.1260064 25554784

[B14] Brucker R. M. Harris R. N. Schwantes C. R. Gallaher T. N. Flaherty D. C. Lam B. A.. (2008). Amphibian chemical defense: antifungal metabolites of the microsymbiont *Janthinobacterium lividum* on the salamander *Plethodon cinereus*. J. Chem. Ecol. 34, 1422–1429. 10.1007/s10886-008-9555-7 18949519

[B15] Castresana J. (2000). Selection of conserved blocks from multiple alignments for their use in phylogenetic analysis. Mol. Biol. Evol. 17, 540–552. 10.1093/oxfordjournals.molbev.a026334 10742046

[B16] Cretoiu M. S. Korthals G. W. Visser J. H. M. van Elsas J. D. (2013). Chitin amendment increases soil suppressiveness toward plant pathogens and modulates the actinobacterial and oxalobacteraceal communities in an experimental agricultural field. Appl. Environ. Microbiol. 79, 5291–5301. 10.1128/AEM.01361-13 23811512 PMC3753968

[B17] Dalia A. B. Lazinski D. W. Camilli A. (2014). Identification of a membrane-bound transcriptional regulator that links chitin and natural competence in *Vibrio cholerae*. Mbio 5, e01028–e01013. 10.1128/mBio.01028-13 24473132 PMC3903286

[B18] De Ley J. Segers P. Gillis M. (1978). Intrageneric and intergeneric similarities of *Chromobacterium* and *Janthinobacterium* ribosomal ribonucleic-acid cistrons. Int. J. Syst. Bacteriol. 28:154 10.1099/00207713-28-2-154

[B19] Edgar R. C. (2004). MUSCLE: multiple sequence alignment with high accuracy and high throughput. Nucleic Acids Res. 32, 1792–1797. 10.1093/nar/gkh340 15034147 PMC390337

[B20] Elbing K. Brent R. (2002). Media preparation and baceriological tools. Curr. Protoc. Mol. Biol. 59 Chapter I:Unit 1.1, 1.1.1–1.1.7. 10.1002/0471142727.mb0101s59 18265292

[B21] Ellinger D. Naumann M. Falter C. Zwikowics C. Jamrow T. Manisseri C.. (2013). Elevated early callose deposition results in complete penetration resistance to powdery mildew in Arabidopsis. Plant Physiol. 161, 1433–1444. 10.1104/pp.112.211011 23335625 PMC3585607

[B22] Frey-Klett P. Burlinson P. Deveau A. Barret M. Tarkka M. Sarniguet A. (2011). Bacterial-fungal interactions: hyphens between agricultural, clinical, environmental, and food microbiologists. Microbiol. Mol. Biol. Rev. 75, 583–609. 10.1128/MMBR.00020-11 22126995 PMC3232736

[B23] Gams W. Hoekstra E. S. Aptroot A. (1998). CBS Course of Mycology. Delft: Centraalbureau vorr Schimmelcultures Baarn.

[B24] Gan H. Y. Gan H. M. Savka M. A. Triassi A. J. Wheatley M. S. Smart L. B.. (2014). Whole-genome sequences of 13 endophytic bacteria isolated from shrub willow (*salix*) grown in Geneva, New York. Genome Announc. 2:e00288–14. 10.1128/genomeA.00288-14 24812212 PMC4014680

[B25] García-Alcalde F. Okonechnikov K. Carbonell J. Cruz L. M. Götz S. Tarazona S.. (2012). Qualimap: evaluating next-generation sequencing alignment data. Bioinformatics 28, 2678–2679. 10.1093/bioinformatics/bts503 22914218

[B26] Goo E. Kang Y. Kim H. Hwang I. (2010). Proteomic analysis of quorum sensing-dependent proteins in *Burkholderia glumae*. J. Proteome Res. 9, 3184–3199. 10.1021/pr100045n 20408571

[B27] Gooday G. W. (1990). The ecology of chitin degradation. Adv. Microb. Ecol. 11, 387–430. 10.1007/978-1-4684-7612-5_10

[B28] Goswami R. S. Kistler H. C. (2004). Heading for disaster: *Fusarium graminearum* on cereal crops. Mol. Plant Pathol. 5, 515–525. 10.1111/j.1364-3703.2004.00252.x 20565626

[B29] Graupner K. Lackner G. Hertweck C. (2015). Genome sequence of mushroom soft-rot pathogen *Janthinobacterium agaricidamnosum*. Genome Announc. 3:e00277–15. 10.1128/genomeA.00277-15 25883287 PMC4400430

[B30] Harris R. N. Brucker R. M. Walke J. B. Becker M. H. Schwantes C. R. Flaherty D. C.. (2009). Skin microbes on frogs prevent morbidity and mortality caused by a lethal skin fungus. ISME J. 3, 818–824. 10.1038/ismej.2009.27 19322245

[B31] Henke J. M. Bassler B. L. (2004). Three parallel quorum-sensing systems regulate gene expression in *Vibrio harveyi*. J. Bacteriol. 186, 6902–6914. 10.1128/JB.186.20.6902-6914.2004 15466044 PMC522208

[B32] Higgins D. A. Pomianek M. E. Kraml C. M. Taylor R. K. Semmelhack M. F. Bassler B. L. (2007). The major *Vibrio cholerae* autoinducer and its role in virulence factor production. Nature 450, 883–886. 10.1038/nature06284 18004304

[B33] Hillman K. Gooday G. W. Prosser J. I. (1989). The mineralization of chitin in the sediments of the ythan-estuary, Aberdeenshire, Scotland. Estuar. Coast. Shelf Sci. 29, 601–612. 10.1016/0272-7714(89)90013-9

[B34] Hiraishi A. Shin Y. K. Sugiyama J. (1997). Proposal to reclassify *Zoogloea ramigera* IAM 12670 (P. R. Dugan 115) as *Duganella zoogloeoides* gen. nov., sp. nov. Int. J. Syst. Bacteriol. 47, 1249–1252. 10.1099/00207713-47-4-1249 9336937

[B35] Hornung C. Poehlein A. Haack F. S. Schmidt M. Dierking K. Pohlen A.. (2013). The *Janthinobacterium* sp. HH01 genome encodes a homologue of the *V. cholerae* CqsA and *L. pneumophila* LqsA autoinducer synthases. PLoS ONE 8:e55045. 10.1371/journal.pone.0055045 23405110 PMC3566124

[B36] Jansen C. von Wettstein D. Schäfer W. Kogel K. H. Felk A. Maier F. J. (2005). Infection patterns in barley and wheat spikes inoculated with wild-type and trichodiene synthase gene disrupted *Fusarium graminearum*. Proc. Natl. Acad. Sci. U.S.A. 102, 16892–16897. 10.1073/pnas.0508467102 16263921 PMC1283850

[B37] Kämpfer P. Wellner S. Lohse K. Martin K. Lodders N. (2012). *Duganella phyllosphaerae* sp. nov., isolated from the leaf surface of *Trifolium repens* and proposal to reclassify *Duganella violaceinigra* into a novel genus as *Pseudoduganella violceinigra* gen. nov., comb. nov. Syst. Appl. Microbiol. 35, 278–278. 10.1016/j.syapm.2012.02.001 22169565

[B38] Kielak A. M. Cretoiu M. S. Semenov A. V. Sørensen S. J. van Elsas J. D. (2013). Bacterial chitinolytic communities respond to chitin and pH alteration in soil. Appl. Environ. Microbiol. 79, 263–272. 10.1128/AEM.02546-12 23104407 PMC3536121

[B39] Kim S. J. Shin S. C. Hong S. G. Lee Y. M. Lee H. Lee J.. (2012). Genome sequence of *Janthinobacterium* sp. strain PAMC 25724, isolated from alpine glacier cryoconite. J. Bacteriol. 194, 2096. 10.1128/JB.00096-12 22461541 PMC3318480

[B40] Kobayashi D. Y. Crouch J. A. (2009). Bacterial/fungal interactions: from pathogens to mutualistic endosymbionts. Annu. Rev. Phytopathol. 47, 63–82. 10.1146/annurev-phyto-080508-081729 19400650

[B41] Lackner G. Moebius N. Hertweck C. (2011). Endofungal bacterium controls its host by an hrp type III secretion system. ISME J. 5, 252–261. 10.1038/ismej.2010.126 20720578 PMC3105691

[B42] Langmead B. Salzberg S. L. (2012). Fast gapped-read alignment with Bowtie 2. Nat. Methods 9, 357–359. 10.1038/nmeth.1923 22388286 PMC3322381

[B43] Lassak J. Henche A.-L. Binnenkade L. Thormann K. M. (2010). ArcS, the cognate sensor kinase in an atypical Arc system of *Shewanella oneidensis* MR-1. Appl. Environ. Microbiol. 76, 3263–3274. 10.1128/AEM.00512-10 20348304 PMC2869118

[B44] Leach J. Lang B. R. Yoder O. C. (1982). Methods for selection of mutants and *in vitro* culture of *Cochliobolus heterostrophus*. J. Gen. Microbiol. 128, 1719–1729. 10.1099/00221287-128-8-1719

[B45] Lechner M. Findeiss S. Steiner L. Marz M. Stadler P. F. Prohaska S. J. (2011). Proteinortho: detection of (co-)orthologs in large-scale analysis. BMC Bioinform. 12:124. 10.1186/1471-2105-12-124 21526987 PMC3114741

[B46] Lincoln S. P. Fermor T. R. Tindall B. J. (1999). Janthinobacterium agaricidamnosum sp. nov., a soft rot pathogen of Agaricus bisporus. Int. J. Syst. Bacteriol. 49(Pt 4), 1577–1589. 10.1099/00207713-49-4-1577 10555339

[B47] Lo Scrudato M. Blokesch M. (2012). The regulatory network of natural competence and ransformation of *Vibrio cholerae*. PLoS Genet. 8:e1002778. 10.1371/journal.pgen.1002778 22737089 PMC3380833

[B48] Lo Scrudato M. Blokesch M. (2013). A transcriptional regulator linking quorum sensing and chitin induction to render *Vibrio cholerae* naturally transformable. Nucleic Acids Res. 41, 3644–3658. 10.1093/nar/gkt041 23382174 PMC3616704

[B49] Markowitz V. M. Chen I.-M. A. Palaniappan K. Chu K. Szeto E. Pillay M.. (2014). IMG 4 version of the integrated microbial genomes comparative analysis system. Nucleic Acids Res. 42, D560–D567. 10.1093/nar/gkt963 24165883 PMC3965111

[B50] McTaggart T. L. Shapiro N. Woyke T. Chistoserdova L. (2015). Draft genome of *Janthinobacterium* sp. RA13 isolated from lake Washington sediment. Genome Announc. 3:e01588–14. 10.1128/genomeA.01588-14 25676775 PMC4333675

[B51] Medema M. H. Blin K. Cimermancic P. de Jager V. Zakrzewski P. Fischbach M. A.. (2011). antiSMASH: rapid identification, annotation and analysis of secondary metabolite biosynthesis gene clusters in bacterial and fungal genome sequences. Nucleic Acids Res. 39, W339–W346. 10.1093/nar/gkr466 21672958 PMC3125804

[B52] Mojib N. Philpott R. Huang J. P. Niederweis M. Bej A. K. (2010). Antimycobacterial activity *in vitro* of pigments isolated from Antarctic bacteria. Antonie van Leeuwenhoek, 98, 531–540. 10.1007/s10482-010-9470-0 20556653

[B53] Morohoshi T. Fukamachi K. Kato M. Kato N. Ikeda T. (2010). Regulation of the violacein biosynthetic gene cluster by acylhomoserine lactone-mediated quorum sensing in *Chromobacterium violaceum* ATCC 12472. Biosci. Biotechnol. Biochem. 74, 2116–2119. 10.1271/bbb.100385 20944413

[B54] Mortazavi A. Williams B. A. McCue K. Schaeffer L. Wold B. (2008). Mapping and quantifying mammalian transcriptomes by RNA-Seq. Nat. Methods 5, 621–628. 10.1038/nmeth.1226 18516045 PMC13303166

[B55] Nirenberg H. I. (1981). A simplified method for identifying *Fusarium* spp. occurring on wheat. Can. J. Bot. 59, 1599–1609. 10.1139/b81-217

[B56] Poehlein A. Riegel K. König S. M. Leimbach A. Daniel R. Dürre P. (2015). Genome sequence of *Clostridium sporogenes* DSM 795(T), an amino acid-degrading, nontoxic surrogate of neurotoxin-producing *Clostridium botulinum*. Stand. Genomic Sci. 10, 40. 10.1186/s40793-015-0016-y 26221421 PMC4517662

[B57] Ramsey J. P. Mercurio A. Holland J. A. Harris R. N. Minbiole K. P. (2015). The cutaneous bacterium *Janthinobacterium lividum* inhibits the growth of *Trichophyton rubrum in vitro*. Int. J. Dermatol. 54, 156–159. 10.1111/ijd.12217 23968275

[B58] Reasoner D. J. Geldreich E. E. (1985). A new medium for the enumeration and subculture of bacteria from potable water. Appl. Environ. Microbiol. 49, 1–7. 3883894 10.1128/aem.49.1.1-7.1985 PMC238333

[B59] Sambrook J. A. Russell D. W. (2001). Molecular Cloning: A Laboratory Manual, 3rd Edn. Cold Spring Harbor, NY: Cold Spring Harbor Laboratory Press.

[B60] Schell U. Simon S. Sahr T. Hager D. Albers M. F. Kessler A.. (2016). The α-hydroxyketone LAI-1 regulates motility, Lqs-dependent phosphorylation signalling and gene expression of *Legionella pneumophila*. Mol. Microbiol. 99, 778–793. 10.1111/mmi.13265 26538361

[B61] Schuster M. Lostroh C. P. Ogi T. Greenberg E. P. (2003). Identification, timing, and signal specificity of *Pseudomonas aeruginosa* quorum-controlled genes: a transcriptome analysis. J. Bacteriol. 185, 2066–2079. 10.1128/JB.185.7.2066-2079.2003 12644476 PMC151497

[B62] Seemann T. (2014). Prokka: rapid prokaryotic genome annotation. Bioinformatics 30, 2068–2069. 10.1093/bioinformatics/btu153 24642063

[B63] Shoemaker W. R. Muscarella M. E. Lennon J. T. (2015). Genome sequence of the soil bacterium *Janthinobacterium* sp. KBS0711. Genome Announc. 3:e00689–15. 10.1128/genomeA.00689-15 26089434 PMC4472911

[B64] Simon S. Schell U. Heuer N. Hager D. Albers M. F. Matthias J.. (2015). Inter-kingdom signaling by the *Legionella* quorum sensing molecule LAI-1 modulates cell migration through an IQGAP1-Cdc42-ARHGEF9-dependent pathway. PLoS Pathogens 11:e1005307. 10.1371/journal.ppat.1005307 26633832 PMC4669118

[B65] Smith H. Akiyama T. Foreman C. Franklin M. Woyke T. Teshima H.. (2013). Draft genome sequence and description of *Janthinobacterium* sp. strain CG3, a psychrotolerant Antarctic supraglacial stream bacterium. Genome Announc. 1:e00960–13. 10.1128/genomeA.00960-13 24265494 PMC3837175

[B66] Song C. Schmidt R. de Jager V. Krzyzanowska D. Jongedijk E. Cankar K.. (2015). Exploring the genomic traits of fungus-feeding bacterial genus *Collimonas*. BMC Genomics 16:1103. 10.1186/s12864-015-2289-3 26704531 PMC4690342

[B67] Spirig T. Tiaden A. Kiefer P. Buchrieser C. Vorholt J. A. Hilbi H. (2008). The *Legionella* autoinducer synthase LqsA produces an α-hydroxyketone signaling molecule. J. Biol. Chem. 283, 18113–18123. 10.1074/jbc.M801929200 18411263 PMC2440625

[B68] Stamatakis A. (2014). RAxML version 8: a tool for phylogenetic analysis and post-analysis of large phylogenies. Bioinformatics 30, 1312–1313. 10.1093/bioinformatics/btu033 24451623 PMC3998144

[B69] Stauff D. L. Bassler B. L. (2011). Quorum sensing in *Chromobacterium violaceum*: DNA recognition and gene regulation by the CviR receptor. J. Bacteriol. 193, 3871–3878. 10.1128/JB.05125-11 21622734 PMC3147534

[B70] Stopnisek N. Zühlke D. Carlier A. Barberán A. Fierer N. Becher D.. (2016). Molecular mechanisms underlying the close association between soil *Burkholderia* and fungi. ISME J. 10, 253–264. 10.1038/ismej.2015.73 25989372 PMC4681855

[B71] Surette M. G. Miller M. B. Bassler B. L. (1999). Quorum sensing in *Escherichia coli, Salmonella typhimurium*, and *Vibrio harveyi*: a new family of genes responsible for autoinducer production. Proc. Natl. Acad. Sci. U.S.A. 96, 1639–1644. 10.1073/pnas.96.4.1639 9990077 PMC15544

[B72] Tiaden A. Hilbi H. (2012). α-hydroxyketone synthesis and sensing by *Legionella* and *Vibrio*. Sensors (Basel) 12, 2899–2919. 10.3390/s120302899 22736983 PMC3376566

[B73] Tiaden A. Spirig T. Sahr T. Wälti M. A. Boucke K. Buchrieser C.. (2010). The autoinducer synthase LqsA and putative sensor kinase LqsS regulate phagocyte interactions, extracellular filaments and a genomic island of *Legionella pneumophila*. Environ. Microbiol. 12, 1243–1259. 10.1111/j.1462-2920.2010.02167.x 20148929

[B74] Valdes N. Soto P. Cottet L. Alarcon P. Gonzalez A. Castillo A.. (2015). Draft genome sequence of *Janthinobacterium lividum* strain MTR reveals its mechanism of capnophilic behavior. Stand. Genomic Sci. 10, 110. 10.1186/s40793-015-0104-z 26605004 PMC4657372

[B75] Wagner V. E. Bushnell D. Passador L. Brooks A. I. Iglewski B. H. (2003). Microarray analysis of *Pseudomonas aeruginosa* quorum-sensing regulons: effects of growth phase and environment. J. Bacteriol. 185, 2080–2095. 10.1128/JB.185.7.2080-2095.2003 12644477 PMC151498

[B76] Wheeler T. J. Clements J. Finn R. D. (2014). Skylign: a tool for creating informative, interactive logos representing sequence alignments and profile hidden Markov models. BMC Bioinformatics 15:7. 10.1186/1471-2105-15-7 24410852 PMC3893531

[B77] Wiggins P. J. Smith J. M. Harris R. N. Minbiole K. P. C. (2011). Gut of red-backed salamanders (*Plethodon cinereus*) may serve as a reservoir for an antifungal cutaneous bacterium. J. Herpetol. 45, 329–332. 10.1670/10-231.1

[B78] Williams T. C. Ayrapetyan M. Oliver J. D. (2015). Molecular and physical factors that influence attachment of *Vibrio vulnificus* to chitin. Appl. Environ. Microbiol. 81, 6158–6165. 10.1128/AEM.00753-15 26116670 PMC4542263

[B79] Zdobnov E. M. Apweiler R. (2001). InterProScan - an integration platform for the signature-recognition methods in InterPro. Bioinformatics 17, 847–848. 10.1093/bioinformatics/17.9.847 11590104

[B80] Zhu J. Mekalanos J. J. (2003). Quorum sensing-dependent biofilms enhance colonization in *Vibrio cholerae*. Dev. Cell 5, 647–656. 10.1016/S1534-5807(03)00295-8 14536065

