# Supplementary material for: Molecular Keys to the Janthinobacterium and Duganella spp. Interaction with the Plant Pathogen Fusarium graminearum
Source: Front Microbiol. 2016 Oct 26;7:1668. doi: 10.3389/fmicb.2016.01668 (PMC5080296; doi:10.3389/fmicb.2016.01668)
Supplement: Supplementary file 1 [file Table1.pdf]

TABLE S1. Bacterial strains and plasmids used in this study

Strain/ Plasmid	Properties	Reference
**Strains**		
E. coli DH5α pDrive::*vioABCDE*	Cloning strain, *rec*A1, Δ*lac*Z harbouring the plasmid pDrive::*vioABCDE*, Amp^R	(Hornung et al, 2013)
Janthinobacterium agaricidamnosum	Wiltdype isolate, DSM9628	DSMZ, Braunschweig, Germany
J. lividum	Wiltdype isolate, DSM1522	DSMZ, Braunschweig, Germany
J. sp. HH100, HH102, HH103, HH104, HH106, HH107, MP5059B	Wild type isolates, Amp^R, Tc^S, Gm^S, Kan^S	This work
J. sp. HH102Δ*jqsA*	*jqsA* deletion mutant of HH102 (JAN4_14950)	This work
Duganella phyllosphaerae	Wiltdype isolate, DSM23865	DSMZ, Braunschweig, Germany
D. zoogleoides	Wiltdype isolate, DSM16928	DSMZ, Braunschweig, Germany
D. sp. HH01	Wild type isolate, Amp^R, Tc^R, Gm^S, Kan^S	(Hornung et al, 2013)
D. sp. HH01Δ*jqsA*	*jqsA* deletion mutant of HH01	(Hornung et al, 2013)
D. sp. HH101, HH105	Wild type isolates, Amp^R, Tc^R, Gm^S, Kan^S	This work
**Plasmids**		
pK18mobII_pKOScvm	Plasmid to amplify the mCherry gene	(Schluter et al, 2015)
pNPTS138-R6KT	Suicide vector for generating gene deletion mutants	(Lassak et al, 2010)
pNPTS138-R6KT::*jqsA*UpDs	Suicide vector for generating *jqsA* gene deletion mutants in *J.* sp. HH102, (JAB4_14950)	This work
pTS-21	pET-28a(+) harboring the *lqsA* gene from *Legionella pneumophila* El Tor (VC2740)	(Spirig et al, 2008)
pBBR1MCS-2	Broad host-range vector , km^R	(Kovach et al, 1995)
pBBR1MCS-2::$jqsA_{01}$	pBBR1MCS-2 harboring an extra chromosomal copy of the *jqsA* gene of HH01 (Jab_2c24330)	(Hornung et al, 2013)
pBBR1MCS-2::$jqsA_{102}$	pBBR1MCS-2 harboring an extra chromosomal copy of the *jqsA* gene of HH102 (JAB4_14950)	This work
pBBR1MCS-2::*lqsA*	pBBR1MCS-2 harboring an extra chromosomal copy of the *lqsA* gene of *L. pneumophila* (pTS-21)	This work
pBBR1MCS-2::$cqsA_{Vh}$	pBBR1MCS-2 harboring an extra chromosomal copy of the *cqsA* gene of *V. harveyi*	(Hornung et al, 2013)
pBBR1MCS-2::$cqsA_{Vc}$	pBBR1MCS-2 harboring an extra chromosomal copy of the *cqsA* gene of *V. cholerae*	(Hornung et al, 2013)

pBBR1MCS-2::P-mCherry	mCherry derived from pK18mobII_pKOScvm under the constitutive *lac* promoter	This work
pBBR1MCS-2::mCherry	mCherry derived from pK18mobII_pKOScvm, promoterless and cloned in pBBR1MCS-2	This work
pBBR1MCS-2::pvioHH107+JAI::mCherry	pBBR1MCS-2::mCherry with promoter of the violacein operon of HH107, JAB9_09370, including JAI-1 motif	This work
pBBR1MCS-2::pvioHH107-JAI::mCherry	pBBR1MCS-2::pvioHH107+JAI::mCherry without JAI-1 motif	This work

Abbreviations describing geno- and phenotypes were made according to Bachmann (Bachmann, 1983).

Bachmann BJ 1983. Linkage map of Escherichia coli K-12, edition 7. Microbiol Rev, 47, 180-230

Hornung C, Poehlein A, Haack FS, Schmidt M, Dierking K, Pohlen A, Schulenburg H, Blokesch M, Plener L, Jung K, Bonge A, Krohn-Molt I, Utpatel C, Timmermann G, Spieck E, Pommerening-Roser A, Bode E, Bode HB, Daniel R, Schmeisser C & Streit WR 2013. The Janthinobacterium sp. HH01 genome encodes a homologue of the V. cholerae CqsA and L. pneumophila LqsA autoinducer synthases. PLoS One, 8, e55045.10.1371/journal.pone.0055045

Kovach ME, Elzer PH, Hill DS, Robertson GT, Farris MA, Roop RM, 2nd & Peterson KM 1995. Four new derivatives of the broad-host-range cloning vector pBBR1MCS, carrying different antibiotic-resistance cassettes. Gene, 166, 175-6.0378111995005841 [pii]

Lassak J, Henche AL, Binnenkade L & Thormann KM 2010. ArcS, the cognate sensor kinase in an atypical Arc system of Shewanella oneidensis MR-1. Appl Environ Microbiol, 76, 3263-74

Schluter JP, Czuppon P, Schauer O, Pfaffelhuber P, Mcintosh M & Becker A 2015. Classification of phenotypic subpopulations in isogenic bacterial cultures by triple promoter probing at single cell level. Journal of Biotechnology, 198, 3-14.10.1016/j.jbiotec.2015.01.021

Spirig T, Tiaden A, Kiefer P, Buchrieser C, Vorholt JA & Hilbi H 2008. The Legionella autoinducer synthase LqsA produces an alpha-hydroxyketone signaling molecule. The Journal of biological chemistry, 283, 18113-23.10.1074/jbc.M801929200
