# Supplementary material for: Molecular Keys to the Janthinobacterium and Duganella spp. Interaction with the Plant Pathogen Fusarium graminearum
Source: Front Microbiol. 2016 Oct 26;7:1668. doi: 10.3389/fmicb.2016.01668 (PMC5080296; doi:10.3389/fmicb.2016.01668)
Supplement: Supplementary file 3 [file Table3.PDF]

TABLE S3. Primer used for cloning and mutant construction

Name	Sequence		Annealing temperature	Product size
ΔjqsA$_{102}$-UP-FP-BamHI (oFH120)	GCGGATCCTGTTGGACGCGGTAG TTCCCAC		59 °C	266 bp
ΔjqsA$_{102}$-UP-RP-XbaI (oFH115)	GTTCTAGACGCCTCGCCGGCGAT GTTCAGGTAA			
ΔjqsA$_{102}$-DS-FP-XbaI	GCTCTAGATGACGGATCTCGGCT ACAAC		59 °C	317 bp
ΔjqsA$_{102}$-DS-RP-EcoRI	GCGAATTCCGCTACACCAGCGCC AGCTT			
Pvio$_{107}$+JAI-FP-BamHI	GTGGATCCGCGCCTTGCAGACGA CAACTA		55 °C	300 bp
Pvio$_{107}$+JAI-RP-EcoRI	GGCGAATTCCGTGCTCATACGAA ATTCCT			
Pvio$_{107}$-JAI-FP-XhoI	GACTCGAGATACCCGGGCTATTG TCGGTG		55 °C	1010 bp
mCherry-FP-EcoRI	GCGAATTCATGGTGAGCAAGGGC GAGGA		56 °C	737 bp
mCherry-RP-HindIII	GGCAAGCTTTTACTTGTACAGCTC GTCCAT			
oFH106 (ΔjqsA$_{102}$-UP-FP-proof)	AACGGCAGTTCGAAGGTCAG		59 °C	HH102: 2083 bp<br>HH102Δ*jqsA*: 1344 bp
oFH107 (ΔjqsA$_{102}$-DS-RP-Proof)	AATCTGCACGGCCTGAGCTT			
oFH108 (ΔjqsA$_{102}$-deleted region-FP)	AATCGGCGCAAGCCGTG		60 °C	HH102: 280 bp<br>HH102Δ*jqsA*: 0 bp
oFH109 (ΔjqsA$_{102}$-deleted region-RP)	AGCGACGCCGTGCGGAA			
Δ*jqsA*$_{102}$-comp-FP-XhoI	AGTCTCGAGGCACAATATCGGCA CAACTG		59 °C	1363 bp
Δ*jqsA*$_{102}$-comp-RP-XbaI	GATCTAGAGCTACACCAGCGCCA GCTTGC			
**Gene**	**Forward Primer (5'-3' direction)**	**Reverse Primer (5'-3' direction)**		**Efficiency**
rpoD (Jab_2c27560)	GAACAGCTGGAAGCG TTGAA	CTTGACGTAGGCCTTG GAGTTGTAG		98.4 %
dnaG (Jab_2c27570)	AACCGAAGTACTTGA ATTCTCCCGAAAC	TATAGCCTTCCGTCAC CAGCACATAC		93.4 %

Jab_2c07990	GTCGTCCGACGCGCTCAATA	GATGCTGGCCGGATTCAGGT	94.2 %
Jab_2c08810	ACAATCCTCTGCGCAAGGTCTACTT	CAGGTAGACATCCTCGCCTTGTTC	95.4 %
Jab_2c10590	TCGACCTGGCCGATGTCTAC	TGGCGACGAAGGACACCTTG	90.5 %
Jab_2c16610	ACCTGATCCGCAGCTGGAAC	GGTGTCGGTGACGACCACTT	97.8 %
Jab_2c26580	TGACGTCGAGCGTGACCTTG	CCAGGTTCACGCGCACATTG	86.9 %
Jab_2c35400	TCGGCGATCCGATCGAGTTC	AGTCGAGGTGGCCGAGATTC	97.2 %
rpoD (JAB4_42690)	CCAACTTGCGCCTGGTCATT	GGCGGTACTCGAACTTGTCA	95.1 %
dnaB (JAB4_01060)	TTGACGAGGCCGAGTCGAAG	CTCTGGTTGTCGCGGCTGTA	96.4 %
JAB4_02850	ATCCGCAGCCGCCATCTGTT	AACCTGCCGTCAGCTTGTCC	89.0 %
JAB4_16300	AGATCGCCTATGCCGTGCTG	GCGCTTGTTGACGGTGTCCA	93.8 %
JAB4_20530	TTGCTGCTGCCGATGGTGTC	TGGTAGCCGCGGTCGCATA	89.3 %
JAB4_35690	TTCCTGTCGGCAGTACAGCA	GGCCAGCAAGTTGGTCTTGA	84.3 %
JAB4_35800	TCAAGACGGCGCACGAAGAG	AGCACGGCGATGACGAGGTA	95.7 %
JAB4_42080	CGATCTTCGGCGTGGATTCG	GCGTCTTCCAGCGCGTGATA	88.5 %

Restriction sites are underlined, forward primer are abbreviated FP and reverse primer RP. DS refers to downstream and UP to upstream.
