# Supplementary material for: Molecular Keys to the Janthinobacterium and Duganella spp. Interaction with the Plant Pathogen Fusarium graminearum
Source: Front Microbiol. 2016 Oct 26;7:1668. doi: 10.3389/fmicb.2016.01668 (PMC5080296; doi:10.3389/fmicb.2016.01668)
Supplement: Supplementary file 4 [file Table4.PDF]

TABLE S4. JAI-1 motif and fold change of regulated genes in HH01 and HH102

Locus tag	Predicted function	JAI-1 motif in 5' gene region	Log-fold change
**HH01**			
Jab_1c09990	tetracycline resistance protein	**TTGA** ACACGG **TCAT**	-3.18
Jab_1c18190	putative polysaccharide deacetylase	**CTGA** AACGCA **TCAA**	2.3
Jab_2c07990	lipopolysaccharide biosynthesis protein	**TTGA** TCTGTC **TCAA**	-2.14
Jab_2c08460	Histidine kinase	**TTGA** TATTCC **ACAA**	-2.17
Jab_2c10580	outer membrane protein oprM	**TTTG** ATCTACA **TCAA**	-1.71
Jab_2c16610	Kynureninase	**TTGA** AAAAAA **TCGA**	2.67
Jab_2c19720	TIR domain-containing protein	**TTGA** CATCGA **TCAC**	-2.29
Jab_2c26560	Helix-turn-helix domain-containing protein	**TTGA** CCAGCACA **TCAA**	-7.77
**HH102**			
JAB4_02740	intracellular multiplication and human macrophage-killing	**TTGC** GCAGCG **TCAA**<br>**TTGT** CGTATG **TCAA**	(-3.91)
JAB4_02770	hypothetical protein	**TTGA** GATTAA **TCAG**	(-2.53)
JAB4_02800	hypothetical protein	**CTGA** TTAATC **TCAA**	(-3.73)
JAB4_13500	hypothetical protein	**TTGA** TACCAA **TCAA**	(-3.22)
JAB4_16300	PRC-barrel domain protein	**TTGA** GGCAGA **TCAA**	-2.67
JAB4_23200	bacteriohemerythrin	**TTGA** TCCAGA **TCAT**	-2.58
JAB4_35690	Flp/Fap pilin component	**TTGT** GGCGCG **TCAA**	-2.58
JAB4_42090	hypothetical protein	**TTGC** GGTAAG **TCAA**	(-3.21)

Regulation refers to the fold change of the mutant strains compared to the wild types HH01 and HH102. We considered genes with a fold-change of ≥ 2.0, a likelihood value ≥ 0.9 and a FDR-value of ≤ 0.05 as statistically significant. The parenthesis indicate a non-significant change of gene expression.
