# Supplementary material for: Molecular Keys to the Janthinobacterium and Duganella spp. Interaction with the Plant Pathogen Fusarium graminearum
Source: Front Microbiol. 2016 Oct 26;7:1668. doi: 10.3389/fmicb.2016.01668 (PMC5080296; doi:10.3389/fmicb.2016.01668)
Supplement: Supplementary file 5 [file Table5.PDF]

TABLE S5. JAI-1 motif of the 5' gene regions of the chitinase *chiA,* the AI synthase *jqsA* and the violacein operon *vioA*

Gene	strain	gene number	JAI-1 motif in 5' gene region
chiA	HH01	Jab_2c26490	**TTGG** TACGCG **TCAA**
	HH100, HH102, HH103, HH104, HH107, 5059, 1522	JAB2_53050, JAB4_02140, JAB5_43070, JAB6_08700, JAB9_00320, JAB1_49050, JALI_07700	**TTAG** AGGCCACT **TCAA**
	HH101	JAB3_19810	**TTGG** TATTCG **TCAA**
	HH105	JAB7_54080	**TTGG** TACTCG **TCAA**
	9628	GJA_3469	**TTGA** TACCACT **TCGA**
jqsA	HH01, HH101, HH105	Jab_2c24330, JAB3_22080, JAB7_46330	**TTGA** TCAGCG **TCAA**
	HH100, HH102, HH103, HH104, HH106, HH107	JAB2_10110, JAB4_14950, JAB5_52650, JAB6_32140, JAB8_15610, JAB9_28750	**TTGA** GCTGTA **TCAA**
	5059, 1522	JAB1_37310, JALI_03530	**TTGA** TATACG **TCAA**
	9628	GJA_1197	**TTGA** TGTAGA **TCAA**
	16928	F460DRAFT_03279	**TTGA** TTTTGA **TCAA**
	9628	DUPY_10350	**TTGA** CCTTGA **TCAA**
vioA	HH01	Jab_2c08810	**TTGA** CATTTA **TCAA**
	HH100 - 107, 5059	JAB2_05300, JAB3_04810, JAB4_20530, JAB5_09800, JAB6_20840, JAB7_02320, JAB8_05560, JAB9_09370, JAB1_31660	**TTGA** TATTTA **TCAA**
	1522	JALI_28730	**TTGA** TATTTA **TCAA**

Strain abbreviations: 5059: MP5059B, 1522: *J. lividum*, 9628: *J. agaricidamnosum* and 16928: *D. zoogleoides*. Conserved sequence of JAI-1 motif is written in bold.
