# Supplementary material for: Molecular Keys to the Janthinobacterium and Duganella spp. Interaction with the Plant Pathogen Fusarium graminearum
Source: Front Microbiol. 2016 Oct 26;7:1668. doi: 10.3389/fmicb.2016.01668 (PMC5080296; doi:10.3389/fmicb.2016.01668)
Supplement: Supplementary file 6 [file Image1.PDF]

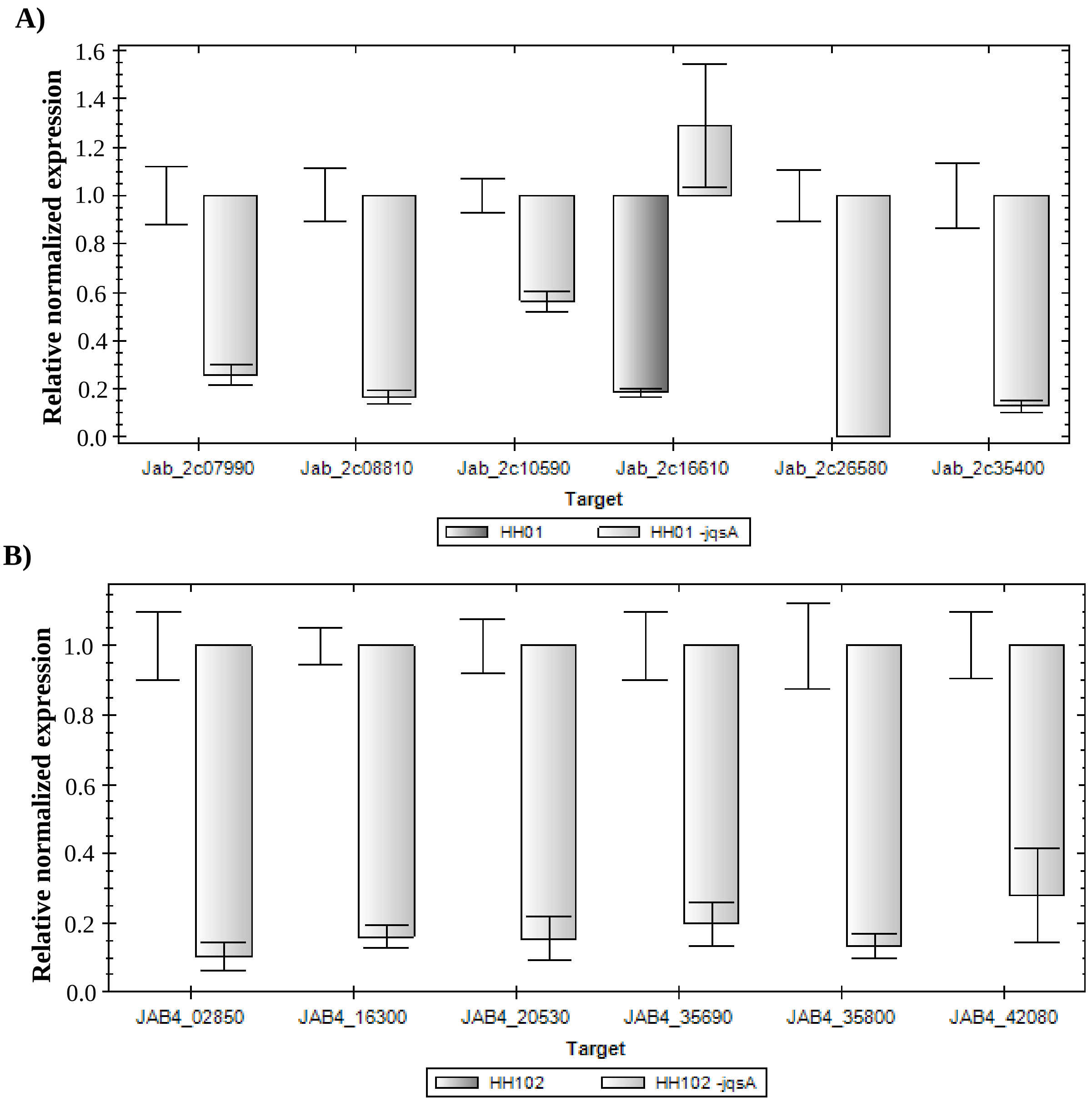

FIGURE S1. qRT-PCR to verify the RNA-seq data of strains A) HH01 / HH01Δ*jqsA* and B) HH102 / HH102Δ*jqsA* at early stationary phase. Shown are the normalized expressions of three technical and three (HH01/HH01Δ*jqsA*) or two (HH102/HH102Δ*jqsA*) biological samples. The graphs are shown with data relative to control using *rpoD* (HH01, HH102), *dnaG* (HH01) and *dnaB* (HH102, Table S2) as house keeping genes.
