# Supplementary material for: Molecular Keys to the Janthinobacterium and Duganella spp. Interaction with the Plant Pathogen Fusarium graminearum
Source: Front Microbiol. 2016 Oct 26;7:1668. doi: 10.3389/fmicb.2016.01668 (PMC5080296; doi:10.3389/fmicb.2016.01668)
Supplement: Supplementary file 7 [file Image2.PDF]

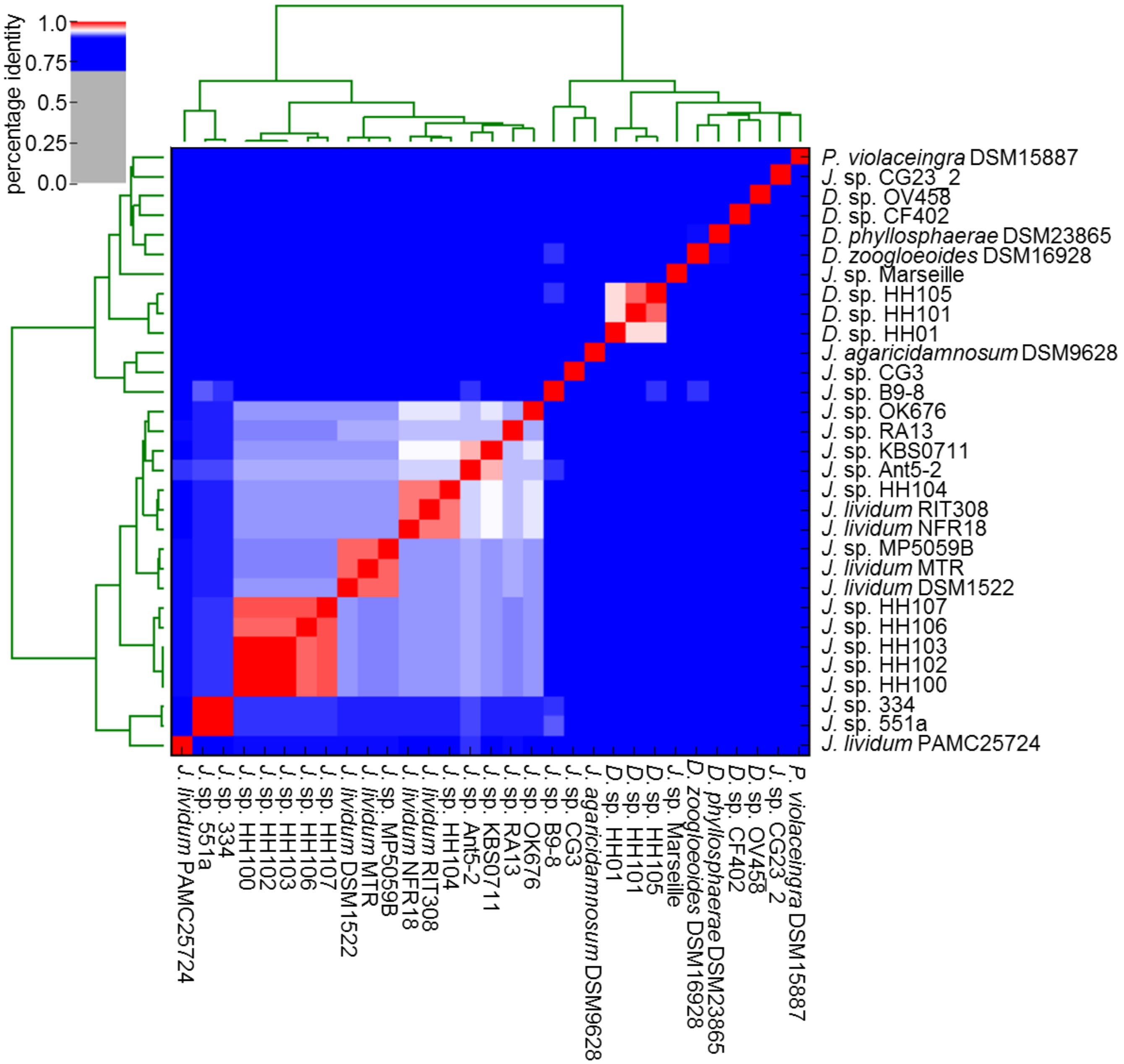

FIGURE S2. ANI percentage identity of sequenced *Duganella*, *Pseudoduganella* and *Janthinobacterium* strains. The ANIm was constructed using genomic data published or extracted from http://www.jgi.doe.gov/ using the IMG software at https://img.jgi.doe.gov/cgi-bin/er/main.cg. Genome information was obtained from the following genome files deposited at GenBank and the respective publications. For strain information consult material and methods, “Bacterial strains and growth”.
