# Supplementary material for: Molecular Keys to the Janthinobacterium and Duganella spp. Interaction with the Plant Pathogen Fusarium graminearum
Source: Front Microbiol. 2016 Oct 26;7:1668. doi: 10.3389/fmicb.2016.01668 (PMC5080296; doi:10.3389/fmicb.2016.01668)
Supplement: Supplementary file 8 [file Image3.pdf]

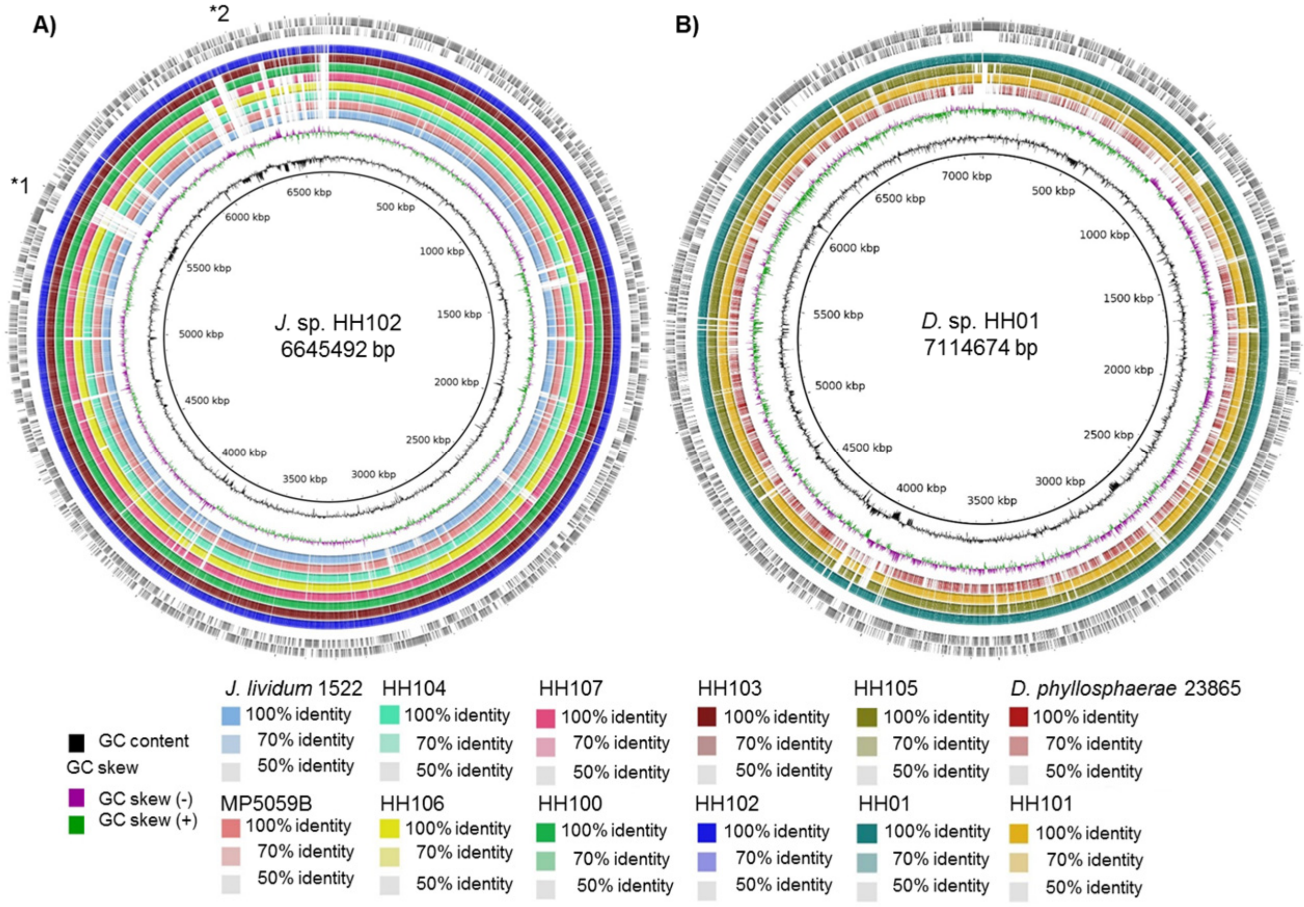

FIGURE S3. Genome maps of the eleven strains sequenced in this study and HH01. The innermost rings indicate the GC content (black) and GC skew (purple/green). The outer rings represent the genomes of the following isolates and two type strains in different colorings: **A)** *J. lividum* (bright blue), MP5059B (bright red), HH104 (turquoise), HH106 (yellow), HH107 (pink), HH100 (green), HH103 (wine red), HH102 (blue) of the OxaI group, related to the genus *Janthinobacterium* and **B)** the OxaII strains, affiliated with the genus *Duganella* HH105 (green), HH01 (petrol), *D. phyllosphaerae* (red) and HH101 (orange). Regions with high levels of divergence are marked with a numbered asterisk. The genome map was created using BRIG (Blast Ring Image Generator; http://sourceforge.net/projects/brig, Alikhan *et al.*, 2011).

Alikhan NF, Petty NK, Ben Zakour NL & Beatson SA 2011. BLAST Ring Image Generator (BRIG): simple prokaryote genome comparisons. BMC Genomics, 12**,** 402.10.1186/1471-2164-12-402
