# Supplementary material for: Molecular Keys to the Janthinobacterium and Duganella spp. Interaction with the Plant Pathogen Fusarium graminearum
Source: Front Microbiol. 2016 Oct 26;7:1668. doi: 10.3389/fmicb.2016.01668 (PMC5080296; doi:10.3389/fmicb.2016.01668)
Supplement: Supplementary file 9 [file Image4.pdf]

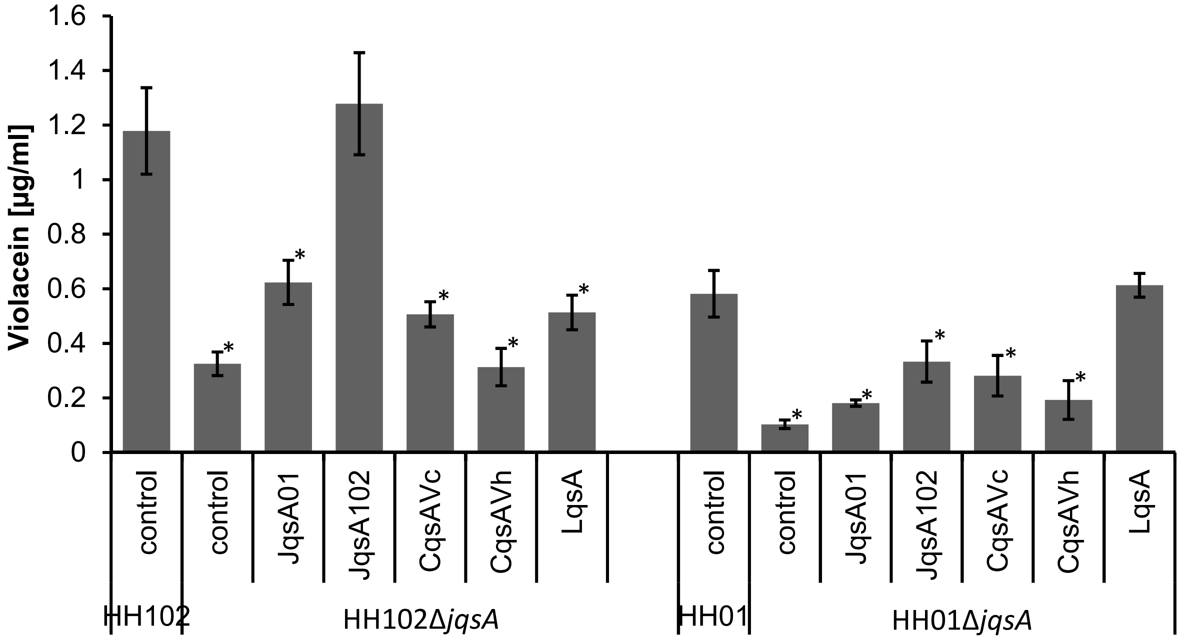

FIGURE S4. Complementation assay of HH102Δ*jqsA* and HH01Δ*jqsA* gene deletion mutants using the AI synthase derived from HH01 (JqsA01), HH102 (JqsA102), *V. cholerae* (CqsAVc), *V. harveyi* (CqsAVh) and *L. pneumophila* (LqsA) cloned in a pBBR1MCS-2. Wild types and gene deletion mutants harboring the empty pBBR1MCS-2 (control) plasmid were used as negative control. Cells were grown for 48 h in R2A media at 22 °C. Experiments were performed three-times. Bars marked with an asterisk are statistically different compared to the corresponding wildtype controls ($P < 0.05$).
